# Supplementary figures and images for: Diet choice: The two-factor host acceptance system of silkworm larvae
Source: PLoS Biol. 2020 Sep 16;18(9):e3000828. doi: 10.1371/journal.pbio.3000828 (PMC7494105; doi:10.1371/journal.pbio.3000828)

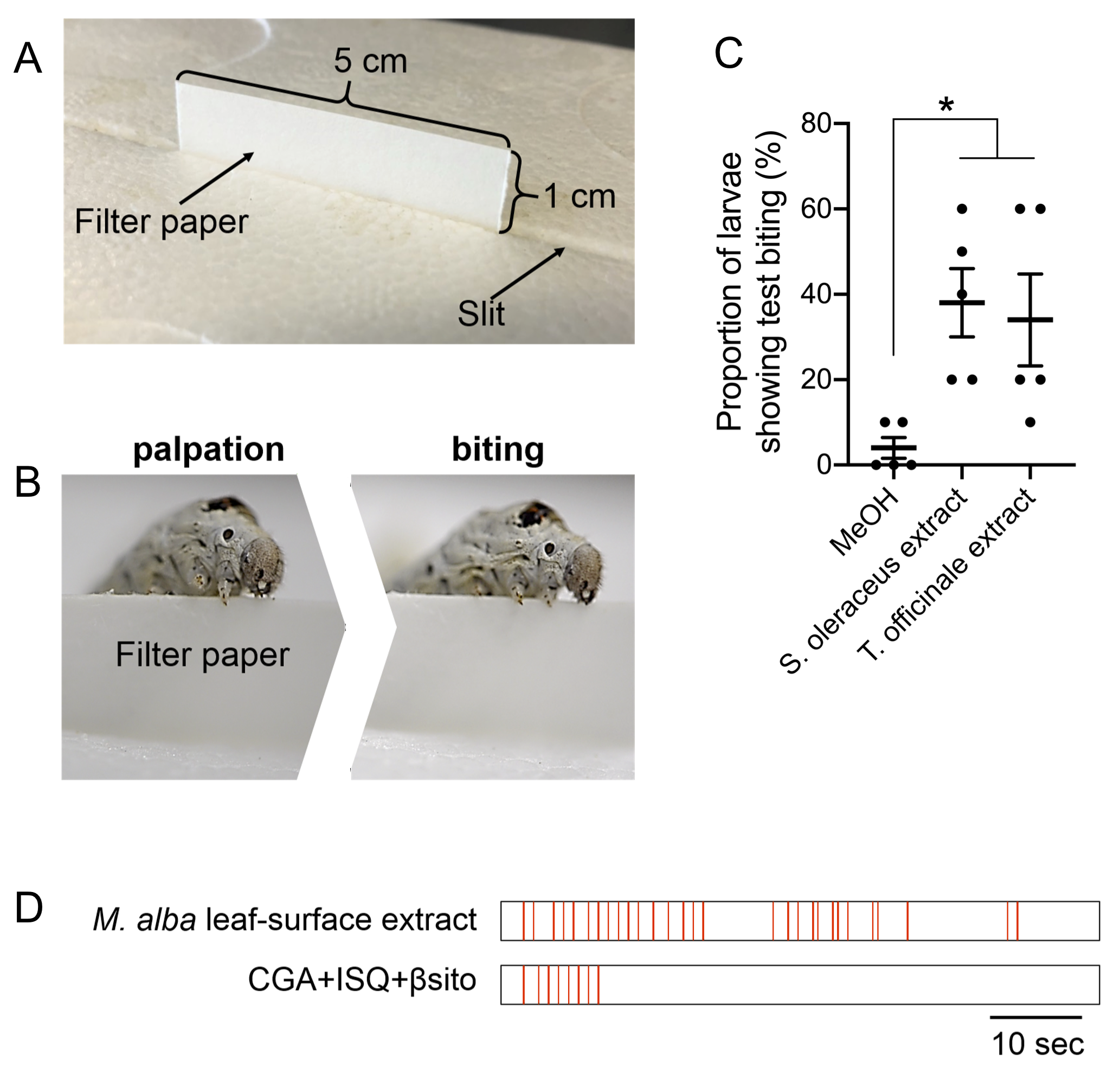

Supplement: S1 Fig — (A) Biting assay using filter paper. Filter paper was inserted into a slit on foamed polystyrene. (B) All larvae palpated at the edge of the filter paper irrespective of treatment (left), and larvae showed test biting of treated filter paper. (C) Proportion of larvae (n = 10) showing test biting of filter paper treated with leaf-surface extracts of 2 edible leaves of S. oleraceus and T. officinale over 1 minute. Experiments were repeated as independent biological replicates (n = 3–5). Data are means ± SE. Statistical analysis was performed using one-way ANOVA followed by Tukey post hoc test. An asterisk indicates a significant difference (*P < 0.05). For numerical raw data, please see S2 Data. (D) Representative raster plots of the timing and duration of biting behavior by larvae using filter paper treated with M. alba leaf-surface extract or a mixture of CGA, ISQ, and βsito. ANOVA, analysis of variance; CGA, chlorogenic acid; ISQ, isoquercitrin; βsito, β-sitosterol. (TIF) [file pbio.3000828.s001.tif]

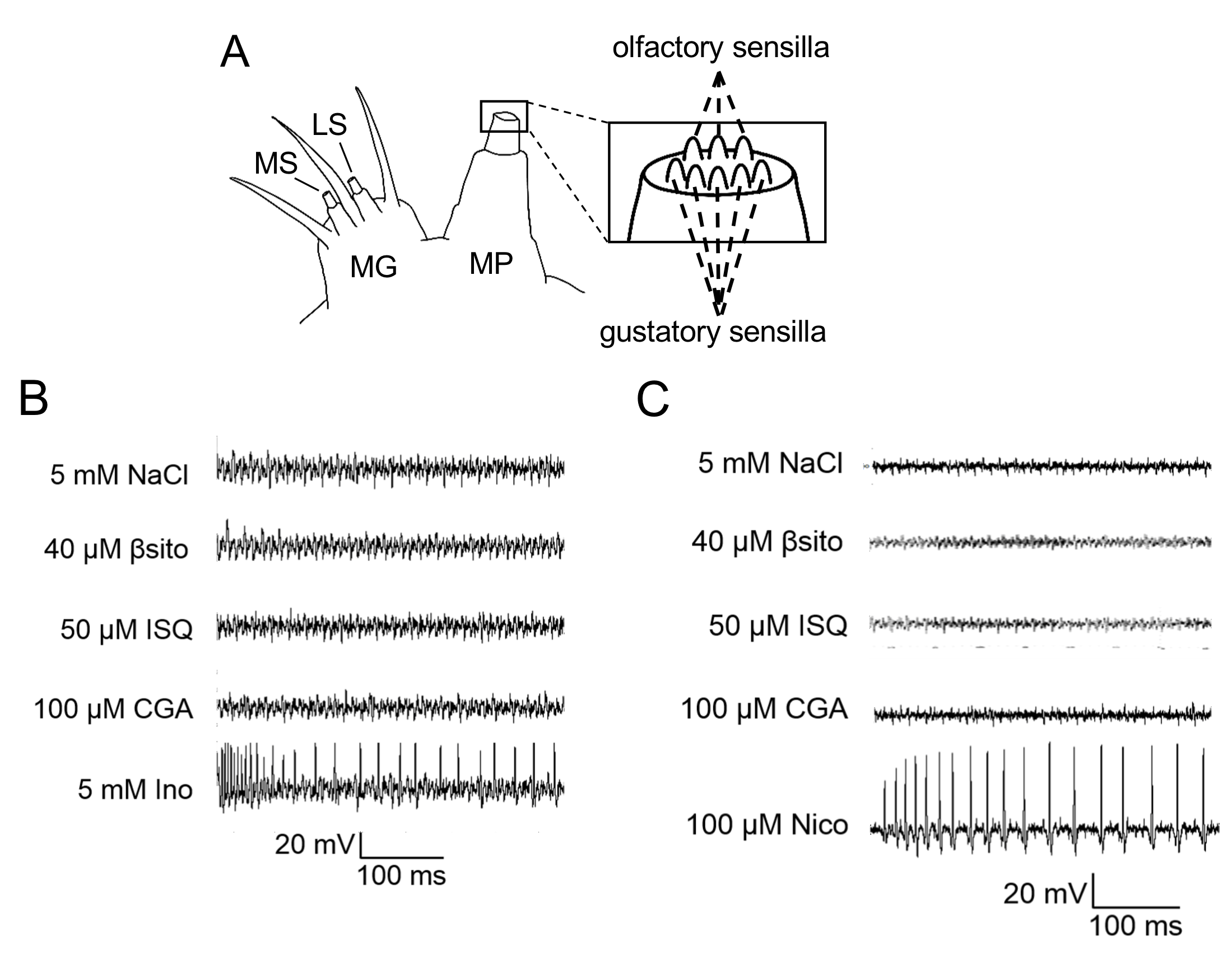

Supplement: S2 Fig — (A) Schematic of sensilla in the MP, which has 8 sensilla (5 putative gustatory and 3 olfactory sensilla). (B and C) Typical electrophysiological recordings from LS (B) and MS (C) in the MG in response to CGA, ISQ, Q3R, and βsito. Ino and Nico were used as positive controls for LS and MS, respectively. CGA, chlorogenic acid; Ino, myo-inositol; ISQ, isoquercitrin; LS, lateral styloconic sensillum; MG, maxillary galea; MP, maxillary palp; MS, medial styloconic sensillum; Nico, nicotine; Q3R, quercetin-3-O-rhamnoside; βsito, β-sitosterol. (TIF) [file pbio.3000828.s002.tif]

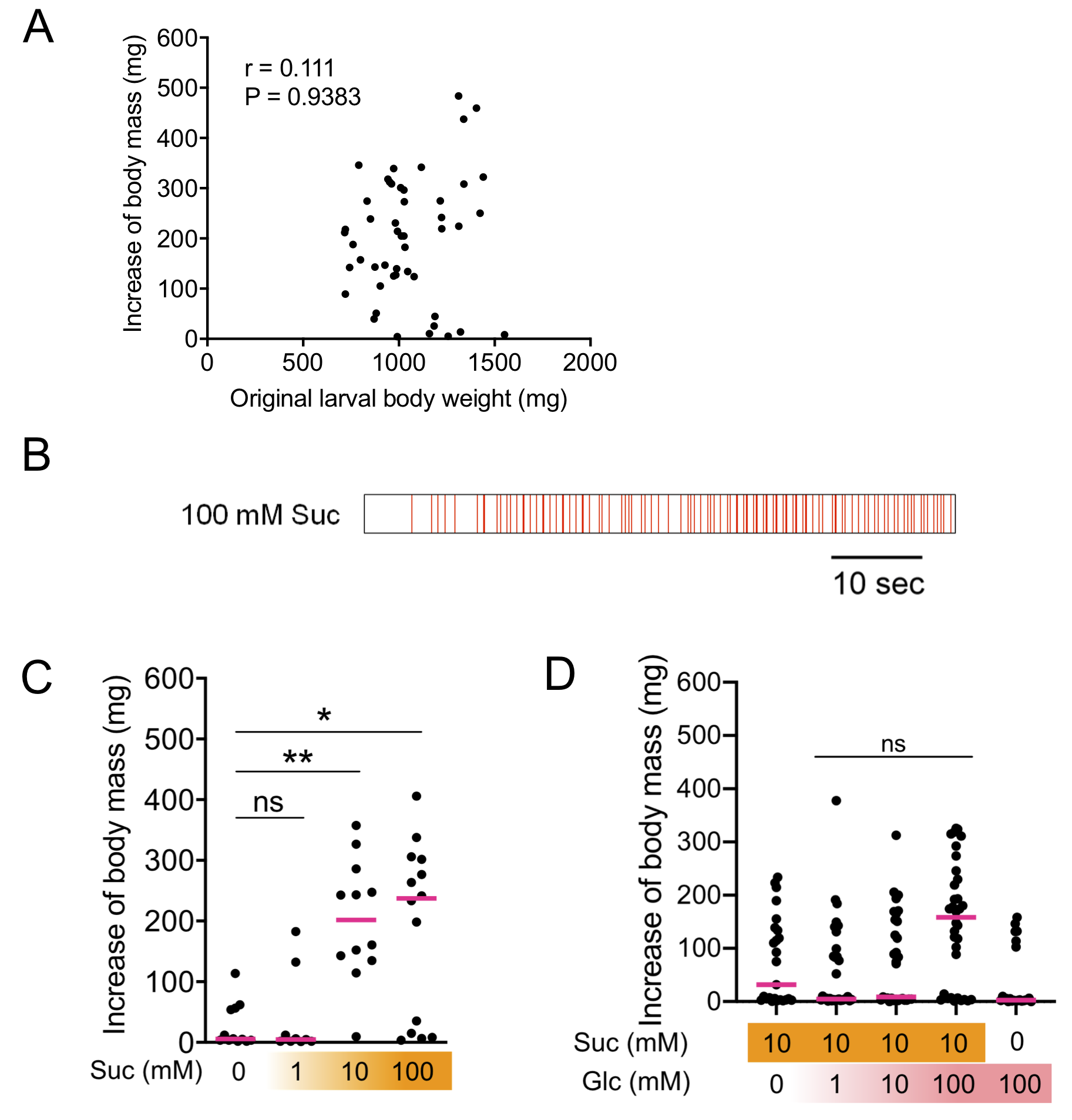

Supplement: S3 Fig — (A) Correlation between the original larval body mass and the increase in body mass after feeding a 100 mM Suc-containing agar-based diet for 3 h. Correlation coefficient (r) by Pearson correlation analysis (n = 51). (B) Representative raster plot of the timing and duration of biting when feeding agar containing 100 mM Suc. (C) Suc-dependent increase in larval weight in MP-ablated larvae after 3 h. (D) An effect of Glu on the increase in larval mass weight by agar-based food-intake assay. Magenta bars denote median. Statistical analysis was performed using Kruskal–Wallis test followed by Dunn test. “ns” indicates no significant difference; an asterisk indicates a significant difference (*P < 0.05; **P < 0.01). For numerical raw data, please see S2 Data. Glc, D-glucose; MP, maxillary palp; Suc, sucrose. (TIF) [file pbio.3000828.s003.tif]

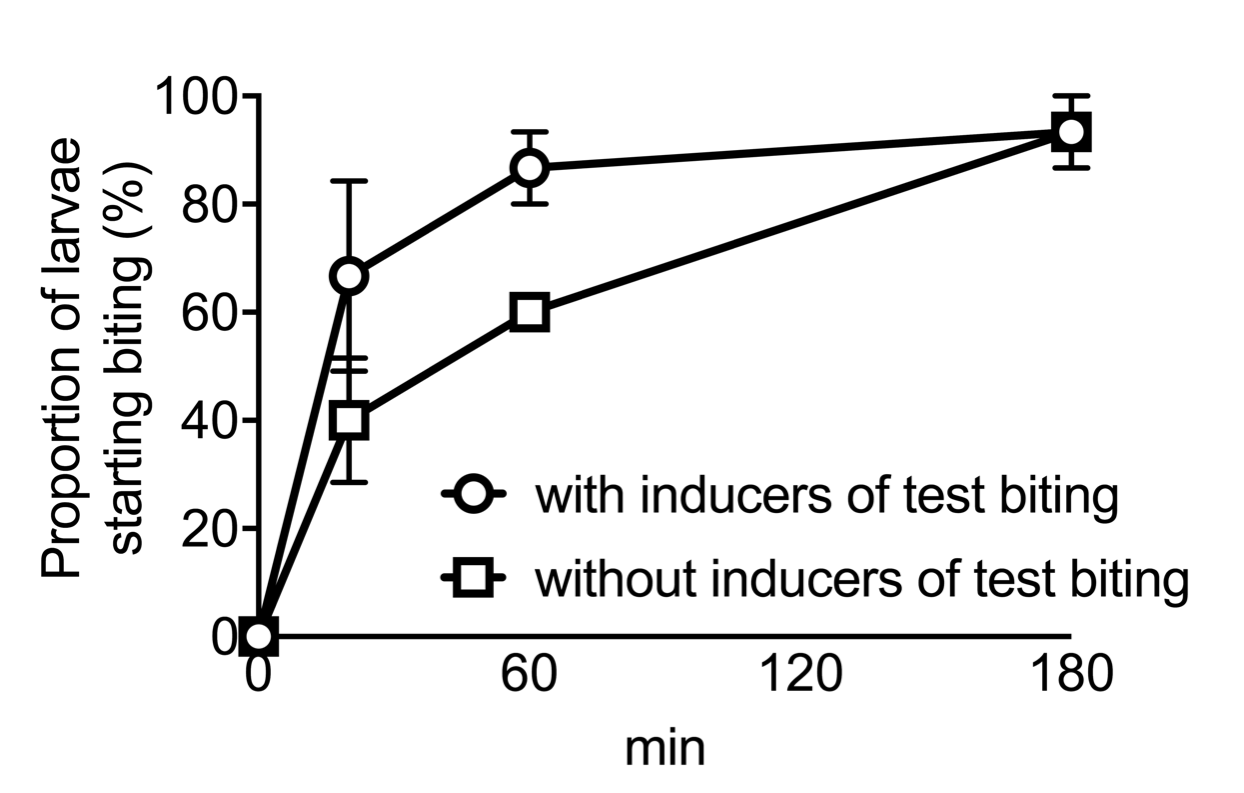

Supplement: S4 Fig — Sugars (10 mM sucrose, 5 mM myo-inositol, and 5 mM D-glucose) were added to the basic agar food (9% cellulose and 1% agar). The following inducers of test biting were added: 100 μM CGA, 1 μM ISQ, and 3 μg/cm2 βsito. Data are from biological triplicate experiments (n = 5); error bars indicate SE. For numerical raw data, please see S2 Data. CGA, chlorogenic acid; ISQ, isoquercitrin; βsito, β-sitosterol. (TIF) [file pbio.3000828.s004.tif]
