# Supplementary material for: Diet choice: The two-factor host acceptance system of silkworm larvae
Source: PLoS Biol. 2020 Sep 16;18(9):e3000828. doi: 10.1371/journal.pbio.3000828 (PMC7494105; doi:10.1371/journal.pbio.3000828)
Supplement: Attachment — Submitted filename: PBIOLOGY-D-20-00139_R2_reviewer1 notes.pdf [file pbio.3000828.s012.pdf]

Manuscript

Click here to access/download;Manuscript;200529 revised manuscript for PBIO.docx

Diet choice: The two-factor host acceptance system of silkworm larvae

Kana Tsuneto[1], Haruka Endo[1,2]*, Fumika Shii[1], Ken Sasaki[3], Shinji Nagata[2] & Ryoichi Sato[1]*

[1]Graduate School of Bio-Applications and Systems Engineering, Tokyo University of Agriculture and Technology, Koganei, Tokyo 184-8588, Japan

[2]Department of Integrated Bioscience, Graduate School of Frontier Sciences, The University of Tokyo, Kashiwa, Chiba 277-8562, Japan

[3]Graduate School of Agriculture, Tamagawa University, Machida, Tokyo 194-8610, Japan

*Correspondence: haruka@edu.k.u-tokyo.ac.jp (H.E.) or ryoichi@cc.tuat.ac.jp (R.S.).

Abstract

Many herbivorous insects are mono- or oligophagous, having evolved to select a limited range of host plants. They specifically identify host-plant leaves using their keen sense of taste. Plant secondary metabolites and sugars are thought to be key chemical cues that enable insects to identify host plants and evaluate their quality as food. However, the neuronal and behavioral mechanisms of host-plant recognition are poorly understood. Here, we report that a two-factor host acceptance system is responsible for host acceptance in larvae of the silkworm *Bombyx mori*, a specialist on several mulberry species. The first step is controlled by a gustatory organ, the maxillary palp (MP). During palpation at the leaf edge, the MP detects trace amounts of leaf-surface compounds, which enables host-plant recognition without biting. Chemosensory neurons in the MP are tuned with ultra-high sensitivity (thresholds of aM to fM) to chlorogenic acid, quercetin glycosides, and β-sitosterol. Only if these three compounds are detected does the larva make a test bite, which is evaluated in the second step. Low-sensitivity chemosensory neurons in another gustatory organ, the maxillary galea (MG), mainly detect sucrose in the leaf sap exuded by test biting, allowing larvae to accept the leaf and proceed to persistent biting (feeding). The two-factor host acceptance system reported here may commonly underlie stereotyped feeding behavior in many phytophagous insects and determine their feeding habits.

Introduction

Diet choice is essential for survival in all organisms. Many herbivorous insects have adapted to a limited range of host plants by overcoming plant defenses against insect herbivory, resulting in mono- and oligophagous insects (so-called specialist insects) [1]. In parallel, specialists are able to precisely distinguish their host plants from other non-host plants in their ecosystem using chemical senses, including gustation and olfaction [2]. In general, gustation is important in determining the acceptance or rejection of a potential food source, whereas olfaction is required when searching for host plants from a distance [3].

Plant-feeding insect larvae exhibit a typical stepwise feeding behavior, comprising foraging, palpation, biting, and swallowing [4,5]. Sensory exploration by palpation of the leaf surface is important for host-plant selection in various plant-feeding insects, such as caterpillars, beetles, and grasshoppers [6-9]. For example, the meadow grasshopper *Chorthippus parallelus* sometimes rejects a diet following palpation using peripheral gustatory organs, without biting [6]. Conversely, leaf-surface extracts of the host plant *Poa annua* induced biting behavior in the migratory locust *Locusta migratoria*, whereas those of non-host plants were rejected [10]. Thus, insects use leaf-surface compounds as chemical cues for host-plant selection, but the neuronal basis for this behavior and to what extent the leaf-surface behavior contributes to whole host-plant selection or leaf choice are poorly understood.

Feeding in insect herbivores is basically an automation controlled by phytochemicals that determine the acceptance or rejection of a leaf based on the balance of feeding stimulants and deterrents contained in plant leaves [3,11,12]. Chemical ecological studies have identified plant secondary metabolites as feeding stimulants and deterrents for many insect hebivores [2]. For example, with regard to the silkworm *Bombyx mori*, a specialist on mulberry leaves, Hamamura and his colleagues identified nonvolatile compounds from mulberry leaves as key signals: isoquercitrin and β-sitosterol as biting factors, and chlorogenic acid as a feeding stimulant that increases feeding amounts in a long-term assay [11,13,14,15]. Thus, feeding stimulants and deterrents in the diet choice have been evaluated by their effects on the number of bites and feeding amounts in most cases. However, these observations do not always reflect the instantaneous behavior of insects that accepts or rejects certain plants [16] and thereby careful investigations along the leaf surface behavior is required for further understanding of role of plant secondary metabolites in host-plant selection.

In the present study, we assigned chemical host cues to taste organs and feeding behaviors to precisely understand the mechanisms underpinning host acceptance in the silkworm. We found that ultra-sensitive chemosensory neurons in the maxillary palp of silkworm larvae detect a set of key mulberry compounds,

including chlorogenic acid, quercetin glycosides, and β-sitosterol, inducing a test bite. Sucrose and *myo*-inositol in leaf saps stimulate lateral sensilla in the maxillary galea to induce continuous biting and acceptance of the leaf. We propose a two-factor host acceptance system, controlled by two peripheral gustatory organs and driven by six phytochemicals, that underlies oligophagy in silkworms.

Results

Two peripheral gustatory organs control two-step biting behaviors in the silkworm. To clarify the mechanism of host acceptance by silkworm larvae, we observed larval feeding of a host leaf from white mulberry *Morus alba*. When a silkworm encounters a leaf, it first palpates the leaf edge using a peripheral gustatory organ known as the maxilla, intermittently bites the edge several times, and finally engages in continuous biting (2–3 times per second) with its head shaking in the dorso-ventral direction along the leaf edge (Fig 1A; S1 Movie). The intermittent biting with palpation and the continuous biting with head-moving are termed test biting and persistent biting [4,5]. We hypothesized that sensing of chemical cues from a *M. alba* leaf via the maxilla induces test biting because test biting always occurs after palpation with the maxilla. The maxilla consists of the maxillary palp (MP) and maxillary galea (MG) (Fig 1B). To assess the roles of MP and MG in induction of test biting, we used MP- or MG-ablated larvae. MP-ablated larvae showed palpation, but no test or persistent biting (Fig 1C and 1D; S2 Movie). MG-ablated larvae showed palpation and test biting, and stopped biting within 1 minute, and did not progress to persistent biting (Fig 1C and 1D; S3 Movie). When we ablated an olfactory organ antenna (AN), the larvae showed palpation, test biting, and persistent biting similar to intact larvae (Fig 1C and 1D; S4 Movie). Note that AN ablation increased the number of bites during first 30 seconds although reasons are unclear. These results indicate that MP and MG are essential for induction of test and persistent biting.

MP recognizes edible leaves by sensing leaf-surface compounds and induces test biting. To assess how such MP- and MG-controlled biting behaviors contribute to host acceptance of the silkworm, we observed feeding behavior towards leaves of various plant species. The silkworm is a specialist for some *Morus* species, including *M. alba*. In addition, the leaves of several Cichorioideae plants of the Asteraceae are consumed in relatively small amounts by silkworm larvae [17,18]. Of the larvae, 88.9 ± 5.3% showed test biting of *M. alba* within 1 minute after reaching the leaf edge, compared to 70.0 ± 10.0 and 63.3 ± 13.3% for two Cichorioideae

plants, *Sonchus oleraceus* and *T. officinale* (Fig 2A). Of the larvae, 80 ± 11.5, 53.3 ± 14.5, and 30 ± 10% proceeded to persistent biting of *M. alba, S. oleraceus,* and *T. officinale*, respectively. In contrast, the larvae had a lower probability of test biting (3–33%) of 12 inedible leaves, which were finally rejected by most of them without persistent biting (Fig 2A and 2B). Thus, the probability of test biting was higher for edible than for inedible leaves and persistent biting was induced only by edible leaves. These results suggest that silkworm larvae select their diet sequentially at two points, before test and persistent biting and that host-plant recognition is largely completed when they make a first bite.

Insects sense leaf-surface compounds during palpation [16]. To elucidate whether leaf-surface compounds induce test biting, we wiped *M. alba* leaves with water or methanol, which markedly decreased the proportion of larvae showing test biting (Fig 2C). Conversely, 76.7 ± 8.8%, 37.5 ± 10.3%, and 36.0 ± 10.8% of the larvae showed test biting of filter paper treated with methanol and leaf-surface extracts of *M. alba*, *S. oleraceus*, and *T. officinale*, respectively (Fig 2D; S1 Fig A–C). MP ablation diminished test biting towards filter paper treated with an extract of *M. alba* leaf-surface (Fig 2D). Furthermore, tip recording of the sensilla in the MP [17] revealed that MP neurons responded to the leaf-surface extract (Fig 2E). These findings indicate that compounds in edible leaf-surface extracts stimulate the MP and trigger test biting.

Test biting requires a set of host-plant compounds detected by ultra-sensitive MP sensory neurons. To identify inducers of test biting, we searched for secondary metabolites in edible leaves of *M. alba*, *T. officinale* and *Lactuca indica* [18,19] in the plant-metabolite database KNApSAcK [20] because secondary metabolites are thought to be key chemical cues for host-plant recognition [2]. The search yielded chlorogenic acid (CGA) and quercetin-3-*O*-rhamnoside (Q3R). CGA reportedly increases the amount of food intake by the silkworm [15]: Q3R is an analog of isoquercitrin (ISQ), which reportedly induces biting by the silkworm [14]. In addition, we focused on β-sitosterol (βsito) because it also reportedly induces biting by the silkworm [13]. We first recorded the responses of MP and MG towards these compounds. Surprisingly, MP responded to the four compounds at the attomolar and femtomolar levels (Fig 3A). In contrast, the MG did not respond to these compounds even at higher concentration (S2 Fig B and C). Next, we assessed whether the four compounds induce test biting. Filter paper, which was treated with each single compound, mixtures of two compounds, and a mixture of ISQ, Q3R, and βsito induced test biting by 20–40% of larvae. In contrast, mixtures of three compounds (CGA+ISQ+βsito, and CGA+Q3R+βsito) and the mixture of all four compounds resulted in a high probability of test biting comparable to the *M. alba* leaf-surface extract (Fig 2D and Fig 3B), but did not

induce persistent biting (S1 Fig D). In agreement with ultra-sensitivity of MP, filter papers treated with extremely dilute mixtures of CGA, ISQ, and βsito still induced test biting to some extent (Fig 3C). Meanwhile, a mixture of fructose, sucrose, glucose, and *myo*-inositol did not induce biting (Fig 3B). These results suggest that a trace amount of set of CGA+ISQ/Q3R+βsito contribute to host recognition and induction of test biting.

MG modulate the amount of food intake by detecting sucrose and *myo*-inositol. Next, we investigated the role of MG in inducing persistent biting. The lateral sensillum (LS) in the MG of the silkworm larvae is involved in recognition of feeding stimulants and has three neurons specifically tuned to glucose, sucrose, and *myo*-inositol respectively at around the millimolar level [21]. Therefore, we hypothesized that sugars in the leaf sap exuded by test biting stimulate the MG and induce persistent biting. As feeding initiation is strictly regulated by test biting, we conducted an agar-based food intake assay using starved larvae [22] to simply evaluate persistent biting. In this assay, starved larvae no longer exert specific preference for mulberry leaves and sometimes randomly bite; this biting substitutes for test biting, and consequently persistent biting occurred in the absence of the inducers of test biting. Alternatively, unlike when feeding on leaves, the MG directly detected high concentrations of compounds at the agar surface during palpation, resulting in induction of persistent biting. Larvae fed agar diet containing sucrose at > 10 mM with showing persistent biting (Fig 4A). The amount of food intake seemed to correlate with the duration of persistent biting. Indeed, a sucrose dose-dependent increase in the body weight was observed in intact and MP-ablated larvae (S3 Fig B and C). The magnitude of the sucrose-induced increase in larval weight was significantly smaller in MG-ablated larvae than in intact larvae (Fig 4A), suggesting an important role of persistent biting via MG in modulating the amount of food intake. Meanwhile, *myo*-inositol and glucose themselves did not induce larval weight increase (Fig 4B; S3 Fig D), whereas *myo*-inositol showed a supplemental effect in the presence of sucrose (Fig 4B) in agreement with a previous study [22]. These results suggest that sucrose and *myo*-inositol contribute to induction of persistent biting by stimulating MG.

Finally, we assessed whether the inducers of test biting (CGA, ISQ, and βsito) and sugars (sucrose, *myo*-inositol, and glucose) resulted in an increase in larval weight similar to that induced by *M. alba* leaf extract and intact leaf. A mixture of 10 mM sucrose, 5 mM *myo*-inositol and 5 mM glucose, similar to the concentrations in *M. alba* leaves [23], resulted in an increase in larval weight in the presence, but not the absence, of a mixture of sugars, similar to a *M. alba* leaf and a leaf-extract-containing agar-based diet (Fig 4C). The test biting induced by CGA+ISQ+βsito accelerated the first bite (S4 Fig), which might cause persistent

biting and consequently increased the total food intake. Therefore, we concluded that these six compounds are major phytochemical drivers of silkworm feeding (Fig 4D).

Discussion

In the present study, we integrated chemical cues, gustatory organs, and biting behaviors to provide new understanding of the mechanisms by which silkworm larvae accept mulberry leaves. Since the 1970s, entomologists have noticed that insects may identify their host plants at the leaf surface, but the mechanisms underlying this phenomenon remained largely unknown. We report here that ultra-sensitive chemosensory neurons in the MP enable silkworm larvae to sense leaf-surface compounds and thus recognize their host plants. Chlorogenic acid, quercetin glycosides, and β-sitosterol were isolated as feeding stimulants for silkworm larvae, but it had been generally thought that these compounds could not explain the preference for mulberry leaves by silkworms, as they are not specific to mulberry. Now, our findings suggest that the combination of the three compounds at the leaf surface explains the preference. Silkworm larvae do not feed on some Moraceae leaves, such as those of fig trees, but will feed on an agar-based diet containing both non-host Moraceae leaves and mulberry leaves [18], suggesting that the non-host Moraceae leaves do not contain feeding deterrents, but rather lack feeding stimulants. The composition of the inducers of test biting at the leaf surface can vary, even among the Moraceae. Alternatively, there may be limited plant species that produce the required combination within the habitat of *Bombyx madarina*, an ancestor of the domesticated silkworm.

Host-plant recognition in larval feeding and adult oviposition seem to employ quite similar systems, driven by multiple host-plant compounds. Some butterflies are reported to require a specific combination of six to ten host-plant compounds to induce egg laying [24-26]. This requirement is very similar to the host-plant acceptance mechanisms that we identified in silkworm larvae feeding. Furthermore, in the swallowtail butterfly *Papilio xuthus*, a specialist feeder on Rutaceae plants, a specific combination of the three neurons detecting multiple oviposition stimulants is responsible for oviposition [27]. The requirement of the combination of three compounds to trigger test biting suggests that different gustatory neurons are responsible for chlorogenic acid, quercetin glycosides, and β-sitosterol, and that the firing of multiple neurons is required to trigger test biting. Thus, insect herbivores may utilize their own combinations of phytochemicals for host-plant recognition in feeding and oviposition.

Our study clarifies an essential role of the MP in host-plant selection and feeding initiation in the silkworm. It was known that the MP has both gustatory and olfactory chemosensory neurons in lepidopteran insects [28], but its role in gustation had previously been poorly studied. We found that the silkworm MP is tuned to several mulberry compounds, but the specificities of more than 20 gustatory neurons in the MP [17] for chemical tastants such as sugars, amino acids, secondary metabolites, salt, and water remain largely uncharacterized. Future work will identify the neurons and receptor molecules that are essential for host-plant recognition. In addition, the possible role of olfaction by the MP in host-plant selection should be addressed. To our knowledge, the sensitivity of the attomolar and femtomolar ranges in gustation is the highest determined to date in insects and other animals. The leaf-surface cuticles are covered by a waxy layer, and therefore the amounts of phytochemicals at the leaf-surface are expected to be low. It is plausible that this ultra-high sensitivity of the MP is necessary to detect trace amounts of inducers of test biting at the leaf surface.

The MG has been shown to play a main role in host-plant selection in lepidopteran insects [3,29]. The medial sensillum (MS) of the silkworm MG is known to respond to feeding deterrents, including coumarin, caffeine, and nicotine, at μM to mM levels [30,31]. Considering the two-factor host acceptance system, a probable role of the MS in the silkworm is to probe for toxic compounds in leaf sap generated by test biting. Most larvae exhibiting test biting on inedible leaves did not proceed to persistent biting, supporting the putative role of the MS in a final check to exclude inedible leaves by detecting feeding deterrents. Numerous information by previous studies on caterpillar neurophysiological responses of the maxillary galea to feeding deterrents [2,3,29] may suggest a similar role of MG in other lepidopteran insects. Note that MG in some lepidopteran species respond to host-plant secondary metabolites [3,29] whereas we did not confirm in the silkworm in this study. In these species, host-plant secondary compounds may stimulate MG and induce persistent biting in combination with sucrose and *myo*-inositol. Meanwhile, insect herbivores often reject a leaf without biting. Glendinning et al. reported that *Manduca sexta* larvae rejected a diet treated with an extract from a non-host plant by sensing it via the MP [32], suggesting the possibility that the MP senses feeding deterrents at the leaf surface with ultra-high sensitivity. Thus, the two-factor host acceptance system likely comprises two-step deterrent effects by the MP and MG in addition to stimulating effects.

By expanding existing models for caterpillars that suggest that feeding is governed by the balance of feeding stimulants and deterrents which stimulate the MG [3,33,34], the two-factor host acceptance system we proposed here may offer a general explanation of host-plant selection in lepidopteran insects. In monophagous/oligophagous caterpillars (i.e. specialists), host-plant recognition by MP is probably conserved and the tuning of neurons in the MP to unique combinations of phytochemicals must reflect a history of insect adaptation to their host plants: In polyphagous caterpillars (i.e. generalists), feeding initiation is expected to be loosely restricted by the MP and thereby rejection of diets by detecting feeding deterrents has a dominant role to select diets as many precious studies have mentioned [2,3]. The stepwise feeding behavior, comprising feeding initiation by recognizing diets and following sustained feeding, is reported in not only phytophagous insects like caterpillars [4,5] and grasshoppers [35] but a wider range of insects. In the fruit fly *Drosophila melanogaster*, the yeast feeding program starts with proboscis extension regulated by the labellum and subsequent sustained feeding is regulated by taste pegs [36]. This suggests that the two-factor system for diet choice, utilizing different taste organs and operating sequentially in time, is largely conserved beyond phytophagous insects. Further comparative studies using other insects will reveal a more concrete picture of the two-factor host acceptance system underpinning their feeding habits.

Materials and Methods

Insects. The silkworm (*B. mori* Kinshu × Showa hybrid) eggs were purchased from Ueda Sanshu Ltd (Nagano, Japan). The silkworms were reared on an artificial diet, Silkmate 2M (Nihon-Nosan Co. Ltd., Kanagawa, Japan) with 16L-8D at 25°C. Larvae were provided with fresh diets every day to synchronize growth.

Biting assay towards leaves and filter paper. Videos of silkworm feeding on leaves and filter paper and filter paper were taken by the D5100 camera (Nikon, Tokyo, Japan) equipped with a telephoto lens AF-S DX Micro Nikkor 85mm (Nikin, Tokyo, Japan). Larvae were used 24 to 48-h after moulting to the fifth instar. To prevent non-specific biting due to starvation, all larvae fed on artificial diets for two minutes before use. For elucidation of the role of chemosensory organs, the maxillary palp, maxillary galea or antenna were quickly removed from a larva with fine forceps after the two-minute feeding. Videos were taken for around 30 to 60 seconds after starting palpation of leaves and filter papers. Fresh host and non-host leaves were collected

within 2 weeks before use around the campuses of Tokyo University of Agriculture and Technology (Fuchu and Koganei, Tokyo, Japan) and stored at 4°C. For preparation of wiped leaves and leaf-surface extract, fresh leaves were gently wiped with kimwipes (Nippon Paper Crecia Co., Tokyo, Japan) wetted with water or methanol. Kimwipes were dried in a drying machine, wet with water or methanol again and put into a centrifugal filter column (Merck Millipore, Darmstadt, Germany), and centrifuged to collect leaf surface extracts. Filter paper (size: 1 cm long, 5 cm wide) was treated with surface extracts from a leaf. A larva moved freely after being put on a leaf and all larvae showed palpation of all tested plant leaves. For the biting assay using commercial compounds, filter paper was impregnated with 100 µl of the stimulant solution. Chlorogenic acid (Nacalai tesque, Kyoto, Japan), isoquercitrin (Extrasynthese, Lyon, France), quercetin-3-*O*-rhamnoside (Extrasynthese) and/or β-sitosterol (Abcam, Cambridge, UK) were dissolved in ethanol, followed by vaporization of ethanol. Filter paper was inserted into a slit on foamed polystyrene (S1 Fig A). A larva was put on the edge of the slit and videotaped. All larvae palpated on filter paper (S1 Fig B). The fraction of larvae showing test or persistent biting was calculated for groups of ten individuals.

An agar-based food intake assay. To determine whether phytochemicals induce persistent biting, we used an agar-based food intake assay [22] with some modification. Agar-based diets basically contain 9% cellulose and 1% agarose in Elix water (Merck Millipore, Tokyo, Japan). Stimulants except for β-sitosterol and cellulose were mixed in advance and added to an agarose solution boiled using the microwave for dissolution. This mixture solution was vortexed well and poured into 90 mm petri-dish (Kanto chemical Co., Inc., Tokyo, Japan). After the agar-based diet solidified, β-sitosterol dissolved in EtOH was applied to the diet followed by EtOH evaporation. For preparation of agar-based diets containing mulberry leaf extract, mulberry leaves were cut into small pieces and dried in a drying machine at 60°C. Dried leaves were crushed into powder. The composition of the mulberry leaf extract diet was 75% mulberry leaf powder (w/v), 1% agarose (w/v), and 24% Elix water (v/v) based on the ratio of dry and wet mass of intact mulberry leaves (data not shown). Three to five larvae were put on each petri-dish and larval mass before and after 3h feeding were measured. Increases of larval mass were regarded as amounts of food intake because no feces were observed after 3h feeding at 25°C. We adopted increases of body mass (mg) as the index of food intake because no significant correlation between amount of food intake and the original body mass was observed ($r = 0.11$, $P > 0.5$) by Pearson’s Correlation Analysis (S3 Fig A).

Tip recording. Tip recordings from all sensilla of MP and the lateral and medial sensilla of MG were conducted to determine whether neurons respond to CGA, ISQ, Q3R and βsito. All eight sensilla in MP (S2 Fig A) were stimulated together. LS and MS in MG have a *myo*-inositol neuron and a deterrent neuron that detects nicotine, respectively [17,30], thereby they were used as positive control for stimulants of LS and MS (S2 Fig B and C). Fifth instar larvae starved until use were used for recording. CGA, ISQ, and Q3R were dissolved and serially-diluted in water whereas βsito was dissolved and diluted in ethanol. In dilution, solutions were stirred well for more than 30 min. We used 5 mM NaCl as an electrolytic solution. Methyl-β-cyclodextrin (βCD) was used as a complexing agent as described in Brown et al. [37] with some modifications. A solution of 2 mg/mL βsito in ethanol was added into 2.5% βCD in 5% NaCl solution so that the final concentration of βsito was 50 μM. The solution was put on a heating block at 80°C until it becomes clear. Tip recording methods were conducted based on Sasaki et al [22]. Stimulants were filled up in glass microelectrodes and a silver wire inside of the electrode was connected to a TastePROBE amplifier (Syntech, Kirchzarten, Germany). The sensilla of MP and the sensilla of MG were capped with the recording electrode using a micromanipulator to stimulate the chemosensory neurons and to record the response simultaneously. As a reference electrode, a fine silver wire insulated to its tip was inserted into the base of the maxilla. Electrical signals were recorded on a computer via a PowerLab 4/25 and analyzed using CHART 5 (ADInstrument, Australia).

Statistics. All statistical analyses were performed using Prism ver.8 (Graph-Pad, La Jolla, USA). We used parametric tests (Fig 2A–D, Fig 3B and 3C, and S1 Fig C) and non-parametric tests (Fig 4A, 4B. 4C and S3 Fig C and D). See S1 Data for more details (*e.g.* exact sample sizes and P value).

Acknowledgements

We thank Toru Shimada and Hiroki Takai (University of Tokyo) for technical assistance in electrophysiological experiments at the early stage of this study; Naoaki Watanabe (Tokyo University of Agriculture and Technology) for assistance in sampling various plants leaves; Yutaka Banno (Kyushu University) for providing fresh *M. alba* leaves; David G. Heckel (Max Planck Institute for Chemical Ecology) and Marian R. Goldsmith (University of Rhode Island) for helpful discussion and critical reading of the manuscript.

References

1. Gatehouse JA. Plant resistance towards insect herbivores: a dynamic interaction. New Phytologist. 2002; 156: 145-169.

2. Pentzold S, Burse A, Boland W. Contact chemosensation of phytochemicals by insect herbivores. Nat Prod Rep. 2017; 34: 478-483.

3. Chapman RF. Contact chemoreception in feeding by phytophagous insects. Annu Rev Entomol. 2003; 48: 455–484.

4. Ma WC. Dynamics of feeding responses in *Pieris brassicae* Linn. as a function of chemosensory input: a behavioral, ultrastructural and electrophysiological study, Meded Landbouwhogesch Wageningen. 1972; 72: 1-162.

5. Devitt BD, Smith JJB. Action of mouthparts during feeding in the dark-sided cutworm, *Euxoa messoria* (Lepidoptera: *Noctuidae*). The Canadian Entomologist. 1985; 117: 343-349.

6. Bernays EA, Chapman RF. Food selection by *Chorthippus parallelus* (Zetterstedt) (Orthoptera: Acrididae) in the field. J Anim Ecol. 1970; 39: 383-394.

7. Chapman RF and de Boer G. Regulatory Mechanisms in Insect Feeding. Chapman & Hall; 1995.

8. Blaney WM, Simmonds MSJ, Ley SV, Katz RB. An electrophysiological and behavioural study of insect antifeedant properties of natural and synthetic drimane-related compounds. Physiol Entomol. 1987; 12: 281-291.

9. Harrison GD. Host-plant discrimination and evolution of feeding preference in the Colorado potato beetle *Leptinotarsa decemlineata*. Physiol Entomol. 1987; 12: 407-415.

10. Bernays EA, Blaney WM, Chapman RF, Cook AG. The ability of *Locusta migratoria* (L.) to perceive plant surface waxes. In: Jermy T, editor. The Host Plant in Relation to Insect Behaviour and Reproduction. Springer-Verlag US; 1976. pp. 35-40.

11. Hamamura Y. Food selection by silkworm larvae. Nature. 1959; 183: 1746-1747.

12. Hirao T, Arai N. On the role of gustatory recognition in host-plant selection by the silkworm, *Bombyx mori*. L. Jpn J Appl Entomol Zool. 1991; 35: 197–206.

13. Hamamura Y, Hayashiya K, Naito K. β-sitosterol as one of the Biting Factors. Nature. 1961; 190: 880-881.

14. Hamamura Y, Hayashiya K, Naito K, Matsuura K, Nishida J. Food selection by silkworm larvae. Nature. 1962; 194: 754.

15. Naito K, Hayashiya K. Studies on the Micro Constituent in Mulberry Leaves. Part VI. Isolation of Chlorogenic Acid. J Agric Biol Chem. 1965; 39: 237-238.

16. Chapman RF, Bernays EA. Insect behavior at the leaf surface and learning as aspects of host plant selection. Experientia. 1989; 45: 215-222.

17. Asaoka K. Ultrastructure of Maxillary Sensilla in the Silkworm, *Bombyx mori*: Differences among Strains? J. Insect Biotechnol Sericol. 2003; 72: 117-125.

18. Uekita K, Ariga Y, Ishida K, Nimura K. Studies on the determinants in the leaves fed exclusively by the silkworm, *Bombyx mori* L [in Japanese]. J Food Sci., Kyoto Women's University. 1971; 26: 1-6.

19. Ohnuma A, Tazima Y. Feeding experiments of polyphagous silkworm larvae on various plant leaves [in Japanese]. J Seric Sci Jpn. 1997; 66: 431-437.

20. Afendi FM, Okada T, Yamazaki M, Hirai-Morita A, Nakamura Y, Nakamura K, et al. KNApSAcK family databases: integrated metabolite-plant species databases for multifaceted plant research. Plant Cell Physiol. 2011; 53: e1.

21. Ishikawa S. Responses of maxillary chemoreceptors in the larva of the silkworm, *Bombyx mori*, to stimulation by carbohydrates. J Cell Physiol. 1963; 61: 99-107.

22. Sasaki K, Ooki Y, Endo Y, Asaoka K. Effects of dietary inositol with sucrose stimulation on chewing and swallowing motor patterns in larvae of the silkworm *Bombyx mori*. Physiol Entomol. 2013; 38: 326-336.

23. Kikuta S, Endo H, Tomita N, Takada T, Morita C, Asaoka K, et al. Characterization of a ligand-gated cation channel based on an inositol receptor in the silkworm, *Bombyx mori*. Insect Biochem Mol Biol. 2016; 74: 12-20.

24. Honda K. Identification of host-plant chemicals stimulating oviposition by swallowtail butterfly, *Papilio protenor*. J Chem Ecol. 1990; 16: 325–337.

25. Ohsugi T, Nishida R, Fukami H. Multi-component system of oviposition stimulants for a Rutaceae-feeding swallowtail butterfly, *Papilio xuthus* (Lepidoptera: Papilionidae). Appl Entmol Zool. 1991; 26: 29–40.

26. Murakami T. Studies on the relationships between host-plant acceptability and plant constituents in host selection by a swallowtail butterfly, *Papilio polytes*. J Grad Sch Biosp Sci. 2003; 42: 58–59.

27. Ryuda M, Calas-List D, Yamada A, Marion-Poll F, Yoshikawa H, Tanimura T, K. et al. Gustatory sensing mechanism coding for multiple oviposition stimulants in the swallowtail butterfly, *Papilio xuthus*. J Neurosci. 2013; 33: 914-924.

28. Schoonhoven LM, Dethier VG. Sensory aspects of host plant discrimination by lepidopterous larvae. Archives Neerlandaises de Zoologie. 2002; 16: 497-530.

29. Schoonhoven LM, van Loon JJA. An inventory of taste in caterpillars: each species its own key. Acta Zoologica Academiae Scientiarum Hungaricae 48 (Suppl. 1). 2002; 215–263.

30. Ishikawa S, Electrical response and function of a bitter substance receptor associated with the maxillary sensilla of the silkworm, *Bombyx mori* L. J Cell Physiol. 1966; 67: 1-11.

31. Asaoka K. Deficiency of gustatory sensitivity to some deterrent compounds in “polyphagous” mutant strains of the silkworm, *Bombyx mori*. J Comp Physiol A. 2000; 186: 1011-1018.

32. Glendinning JI, Valcic S, Timmermann BN. Maxillary palps can mediate taste rejection of plant allelochemicals by caterpillars. J Comp Physiol A. 1998; 183: 35-43.

33. Dethier VG. Electrophysiological studies of gustation in lepidopterous larvae II. Taste spectra in relation to food-plant discrimination. Z Vergl Physiol. 1973; 82:103–134.

34. Simmonds MSJ, Blaney WM. Gustatory codes in lepidopterous larvae. Symp Biol Hung. 1990; 39:17–27.

35. Blaney WM, Chapman RF. The functions of the maxillary palps of Acrididae (Orthoptera). Entomol Exp Appl. 1970; 13: 363-376.

36. Steck K, Walker SJ, Itskov PM, Baltazar C, Moreira JM, Ribeiro C. Internal amino acid state modulates yeast taste neurons to support protein homeostasis in *Drosophila*. eLife. 2018; 7: e31625.

37. Brown AJ, Sun L, Feramisco JD, Brawn MS, Goldstein JL. Cholesterol addition to ER membranes alters conformation of SCAP, the SREBP escort protein that regulates cholesterol metabolism. Mol Cell. 2002; 10: 237-245.

Figure legends

Fig 1. Maxillary palp (MP) and maxillary galea (MG) are responsible for test and persistent biting. (A) Feeding on mulberry leaves by silkworm larvae: (1) Palpation; a silkworm larva first palpates the leaf-surface with its maxilla (MP and MG) for 5–30 seconds. The white arrow and arrowhead indicate MP and MG. (2) Test biting; the larva bites the leaf edge several times intermittently during palpation. (3) Persistent biting; the larva nibbles the leaf edge repeatedly (2–3 times per second) with its head moving in the dorso-ventral direction along the leaf edge. Magenta arrows indicate the direction of head movement. (B) Mouthparts of a silkworm larva. Upper, larval mouthparts including one pair of maxilla. Lower, higher-magnification view of the maxilla. The maxilla consists of the MG and MP. The MG has two gustatory sensilla, the lateral and medial styloconic sensilla (LS and MS). (C) Frequency of biting by intact, MP-ablated, MG-ablated, and antenna (AN)-ablated larvae of mulberry leaves over a 10-second period. Data are means ± SD (n = 5). (D) Representative raster plots of the timing and duration of the biting behavior of larvae in (C). Red and green lines indicate test and persistent bites, respectively.

Fig 2. The MP detects leaf-surface compounds and triggers test biting of edible leaves. (A and B) Proportion of larvae showing test biting (A) and persistent biting (B) of edible and inedible leaves. (C and D) Proportion of larvae showing test biting of intact mulberry leaves or mulberry leaves wiped with sheets of Kimwipe® paper moistened with water or methanol (C) or filter paper treated with methanol or mulberry leaf-surface extract (D). MP + and MP – indicate intact and maxillary palp (MP)-ablated larvae. (A–D) Biting of 10 larvae was observed for 1 minute. Experiments were repeated as independent biological replicates (n = 3–5). Data are means ± SE. The same letters indicate no significant difference ($P > 0.05$) by one-way analysis of variance (ANOVA) followed by Tukey *post hoc* test. (E) Typical spikes from chemosensory sensilla of the MP towards 5 mM NaCl (negative control) and leaf-surface extract (Compounds from a leaf/10 mL).

Fig. 3. A mixture of chlorogenic acid, isoquercitrin/quercetin-3-*O*-rhamnoside, and β-sitosterol induces test biting by stimulating maxillary palp. (A) Typical electrophysiological recordings from the MP in response to chlorogenic acid (CGA), isoquercitrin (ISQ), quercetin-3-*O*-rhamnoside (Q3R), and β-sitosterol. (B and C) Fraction of larvae showing test biting of filter paper treated with CGA, ISQ, Q3R, βsito, and sugars (sucrose, fructose, *myo*-inositol, and glucose) (B) and with an extremely dilute mixture of CGA, ISQ, and βsito (C). Filter paper was treated with 100 µL of 1 mM stimulant solution (100 nmol of each compound/5 cm^2) (B) or a dilution series of the CGA, ISQ, and βsito mixture (C). Biting by 10 larvae was observed for

1 minute. Experiments were repeated as independent biological replicates (n = 3–5). Data are means ± SE. Statistical analysis was performed using one-way analysis of variance (ANOVA) followed by Tukey *post hoc* test. The same letters indicate no significant difference ($P > 0.05$). An asterisk indicates a significant difference (*$P < 0.05$; ***$P < 0.001$; ****$P < 0.0001$).

Fig. 4. Sucrose induces persistent biting, which are modulated by *myo*-inositol and inducers of test biting. (A-C) Effects of sucrose (Suc) (A), *myo*-inositol (Ino) (B), and inducers of test biting, chlorogenic acid (CGA), isoquercitrin (ISQ) and β-sitosterol (βsito) (C) on the increase in larval body mass by agar-based food intake assay. Fifth-instar larvae were starved for 3 days beginning at the end of molting. The body mass of the three larvae increased after feeding of the agar-based diet (9% cellulose and 1% agar) for 3 hours. Magenta bars denote median. Statistical analysis was performed using Kruskal–Wallis test followed by Dunn test (A and B) and Mann-Whitney *U*-test (C). “ns” indicates no significant difference; an asterisk indicates a significant difference (*$P < 0.05$; **$P < 0.01$; ****$P < 0.0001$). MG + and MG – indicate intact and maxillary galea (MG)-ablated larvae. (D) Proposed model of host recognition and acceptance by the silkworm.

Supplementary Materials

S1 Fig. Biting assay using filter paper. (A) Biting assay using filter paper. Filter paper was inserted into a slit on foamed polystyrene. (B) All larvae palpated at the edge of the filter paper irrespective of treatment (left) and larvae showed test biting of treated filter paper. (C) Proportion of larvae (n = 10) showing test biting of filter paper treated with leaf-surface extracts of two edible leaves of *S. oleraceus* and *T. officinale* over 1 minute. Experiments were repeated as independent biological replicates (n = 3–5). Data are means ± SE. Statistical analysis was performed using one-way analysis of variance (ANOVA) followed by Tukey *post hoc* test. An asterisk indicates a significant difference (*$P < 0.05$). (D) Representative raster plots of the timing and duration of biting behavior by larvae using filter paper treated with *M. alba* leaf-surface extract or a mixture of chlorogenic acid (CGA), isoquercitrin (ISQ), and β-sitosterol (βsito).

S2 Fig. Tip recording of sensilla in the maxillary galea (MG). (A) Schematic of sensilla in the maxillary palp (MP), which has eight sensilla (five putative gustatory and three olfactory sensilla). (B and C) Typical electrophysiological recordings from lateral sensilla (LS) (B) and medial sensilla (MS) (C) sensilla in the MG in response to chlorogenic acid (CGA), isoquercitrin (ISQ), quercetin-3-*O*-rhamnoside (Q3R), and β-sitosterol (βsito). *myo*-inositol (Ino) and nicotine (Nico) were used as positive controls for LS and MS, respectively.

S3 Fig. Supplemental agar-based food-intake assay. (A) Correlation between the original larval body mass and the increase in body mass after feeding a 100 mM sucrose (Suc)-containing agar-based diet for 3 hours. Correlation coefficient (r) by Pearson correlation analysis (n = 51). (B) Representative raster plot of the timing and duration of biting when feeding agar containing 100 mM sucrose. (C) Sucrose-dependent increase in larval weight in maxillary palp (MP)-ablated larvae after 3 hours. (D) An effect of glucose (Glu) on the increase in larval mass weight by agar-based food intake assay. Magenta bars denote median. Statistical analysis was performed using Kruskal–Wallis test followed by Dunn test. “ns” indicates no significant difference; an asterisk indicates a significant difference (*$P < 0.05$; **$P < 0.01$).

S4 Fig. Effect of a mixture of inducers of test biting on increase in proportion of feeding larvae. Sugars (10 mM sucrose, 5 mM *myo*-inostiol, and 5 mM glucose) were added to the basic agar food (9% cellulose and 1% agar). The following inducers of test biting were added: 100 µM chlorogenic acid, 1 µM isoquercitrin, and 3 µg/cm^2 β-sitosterol. Data are from biological triplicate experiments (n = 5); error bars indicate SE.

S1 Movie. Biting behavior of an intact larva towards a mulberry leaf.

S2 Movie. Biting behavior of a maxillary palp (MP)-ablated larva towards a mulberry leaf.

S3 Movie. Biting behavior of a maxillary galea (MG)-ablated larva towards a mulberry leaf.

S4 Movie. Biting behavior of an antenna (AN)-ablated larva towards a mulberry leaf.

S1 Data. Statistic information used in this study.

S2 Data. Raw data used in this study.

Figure1

Click here to access/download;Figure;Fig 1.tiff

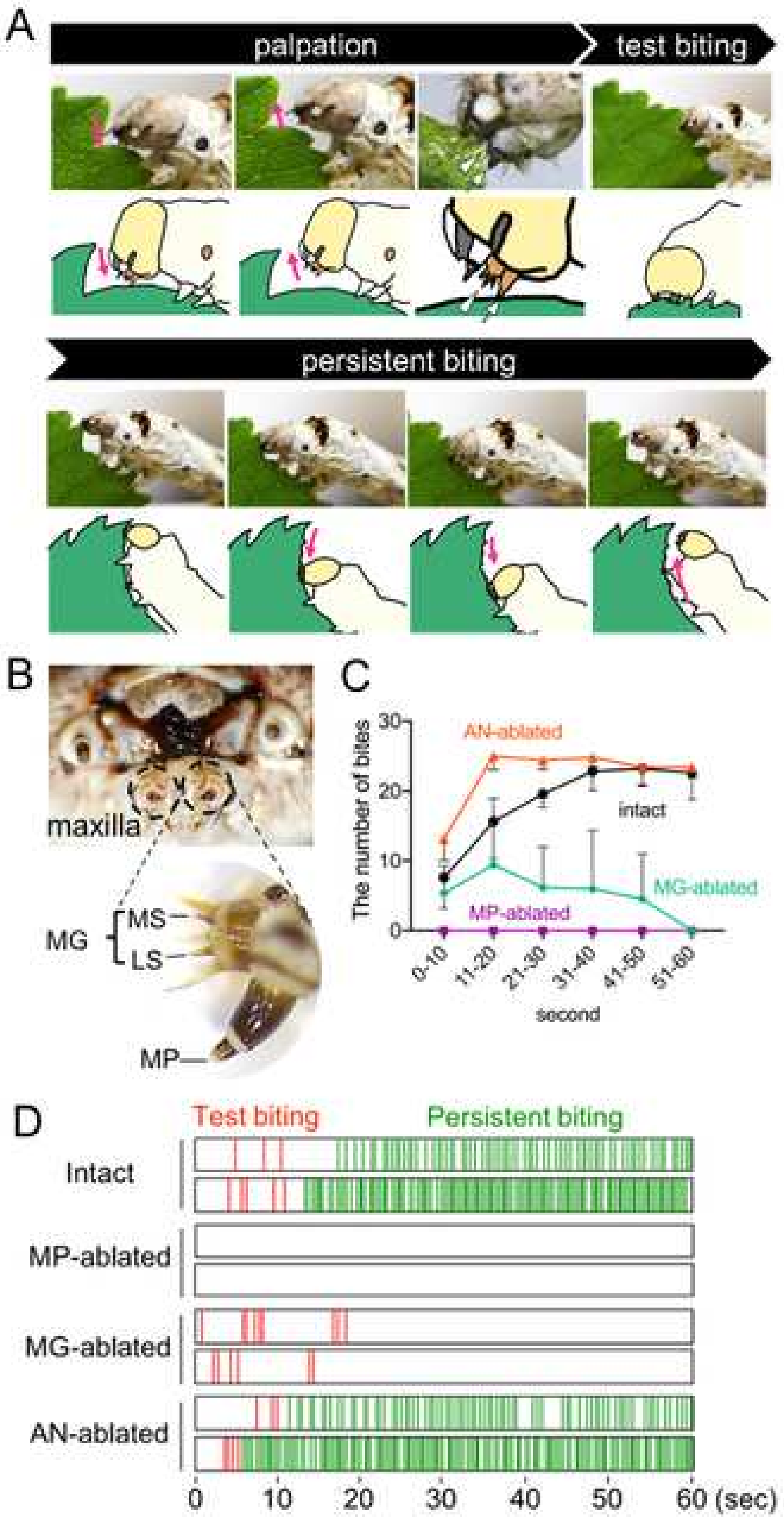

 Click here to access/download;Figure;Fig 2.tiff

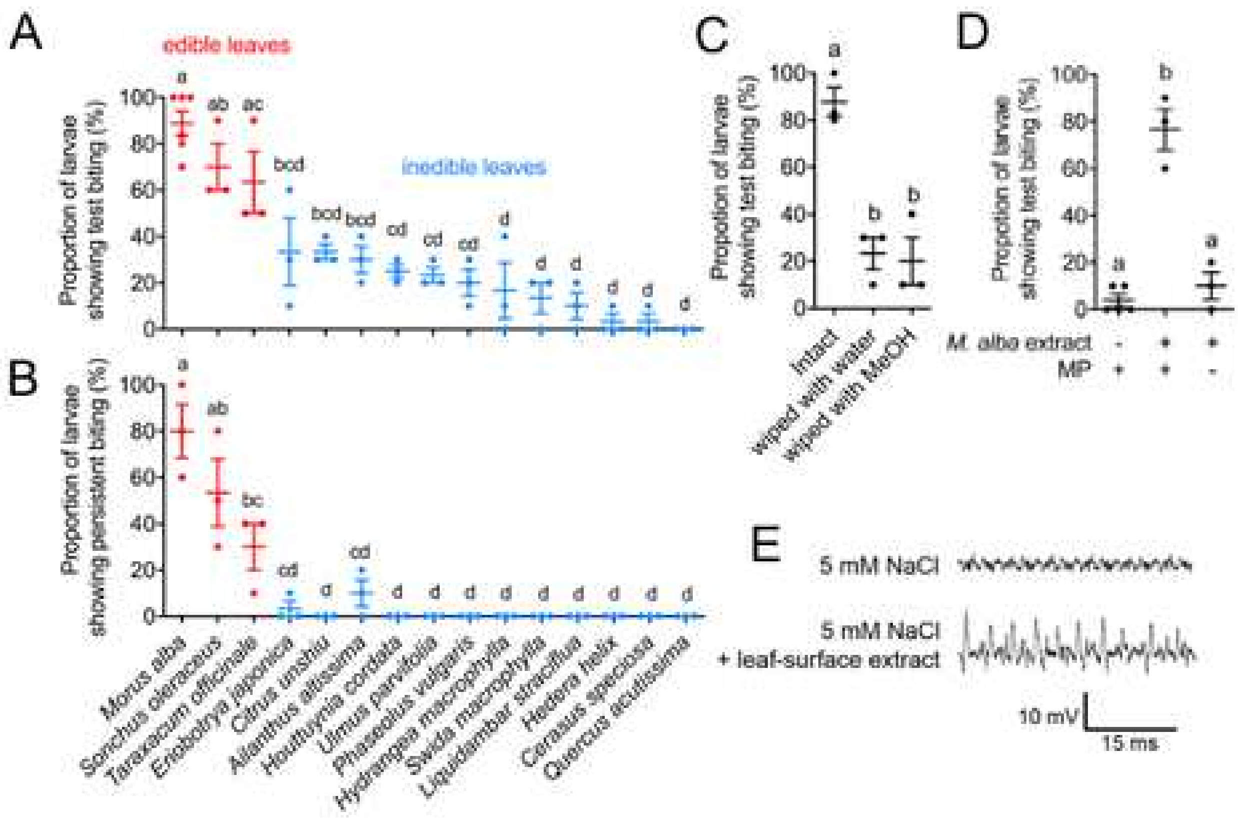

 Click here to access/download;Figure;Fig 3.tiff

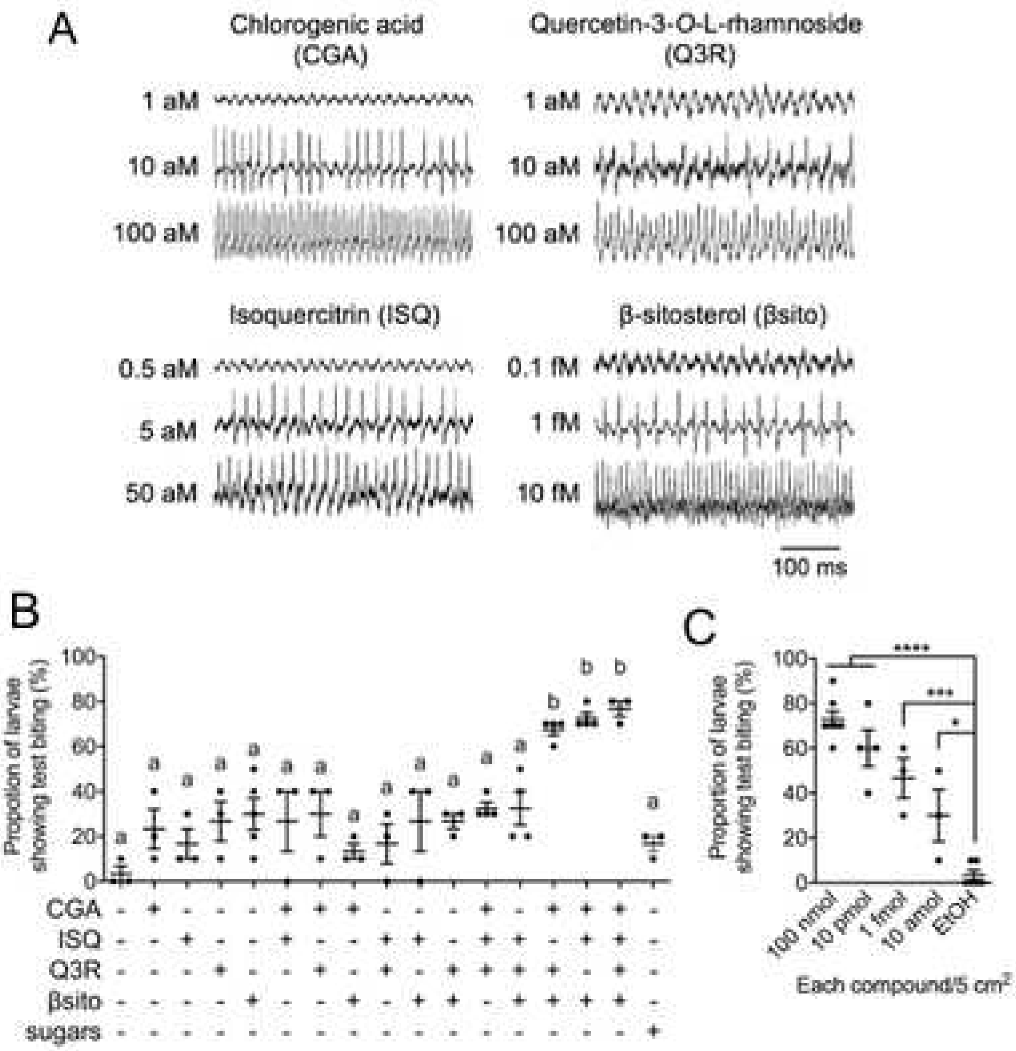

Figure4

Click here to access/download;Figure;Fig 4.tiff

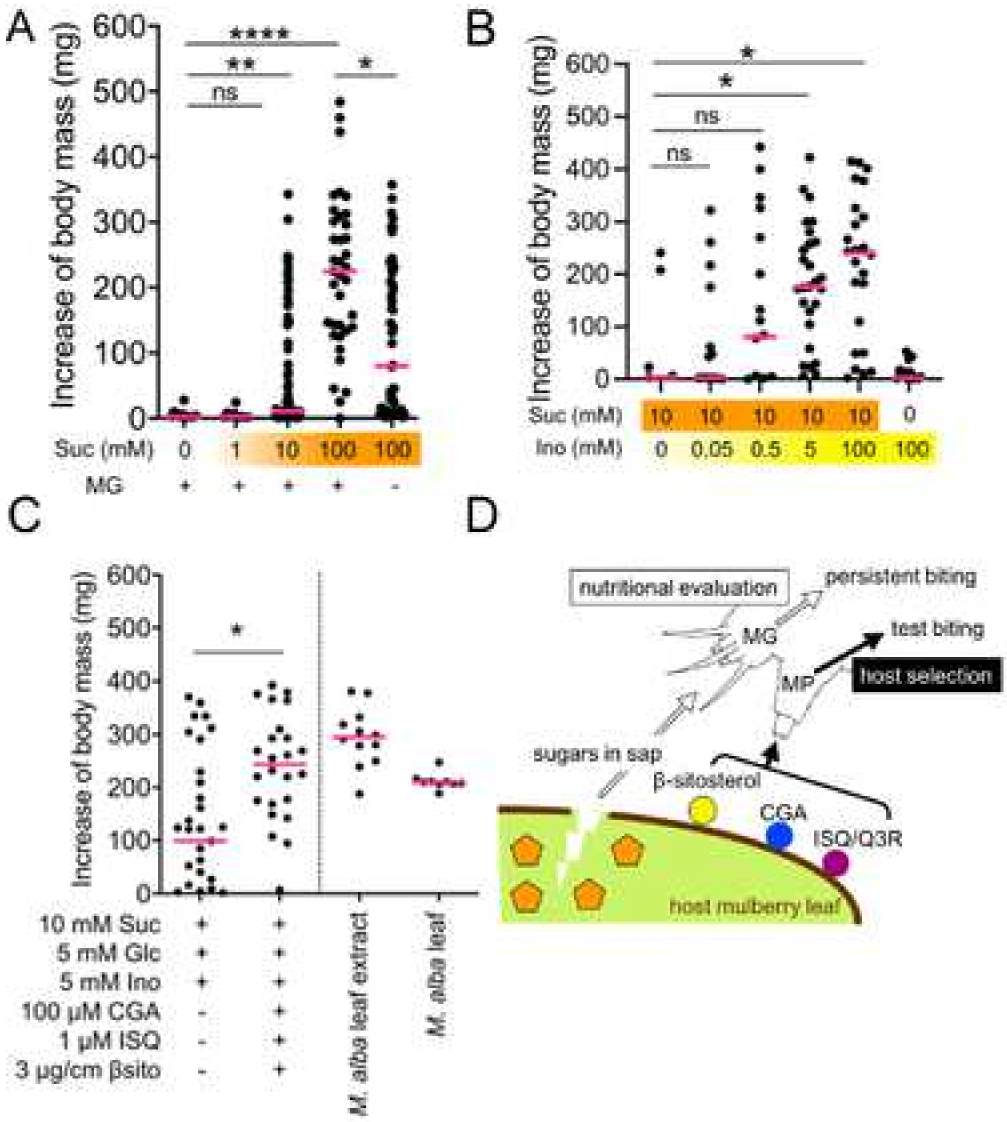

Video S1

Click here to access/download
Supporting Information
Movie S1 intact.mp4

Video S2

Click here to access/download
Supporting Information
Movie S2 MP-ablated.mp4

Video S3

Click here to access/download
Supporting Information
Movie S3 MG-ablated.mp4

Video S4

Click here to access/download
Supporting Information
Movie S4 AN-ablated.mp4

Data S1

Click here to access/download
Supporting Information
Data S1 Statistic information.xlsx

Data S2

Click here to access/download

Supporting Information

Data S2 Raw Data.xlsx

S1 Fig

Click here to access/download
Supporting Information
S1 Fig.tiff

S2 Fig

Click here to access/download
Supporting Information
S2 Fig.tiff

S3 Fig

Click here to access/download
Supporting Information
S3 Fig.tiff

S4 Fig

Click here to access/download
Supporting Information
S4 Fig.tiff

Click here to access/download
Related Manuscript
200529 track-changes revised manuscript for PBIO.docx
