# Supplementary material for: Diet choice: The two-factor host acceptance system of silkworm larvae
Source: PLoS Biol. 2020 Sep 16;18(9):e3000828. doi: 10.1371/journal.pbio.3000828 (PMC7494105; doi:10.1371/journal.pbio.3000828)
Supplement: Attachment — Submitted filename: 200528 response to reviewers comment PBIO.docx [file pbio.3000828.s011.docx]

---------------------------------------------------------------

Reviewers’ comments

Rev. 1:
This manuscript reports an interesting study of neurophysiological and behavioral mechanisms of host-plant acceptance by larval silkworms. As far as I know, the identification of the relative roles of the maxillary palps and galeae in the process of host acceptance represents a new finding. Previous work had mostly focused on particular subsets of the taste system, such as the medial or lateral sensilla of the galeae, or behavioral mechanisms by themselves. And limited work had been done on taste receptors of the maxillary palps. In the present manuscript, the coupling of the electrophysiological work on the taste system encompassing the palps and galeae with behavioral assays was especially refreshing and insightful. The authors conclude that the silkworm accepts a host plant via a "two-factor authentication system" in that stimulation of the taste cells of the maxillary palps first cause biting of the plant, and, second, stimulation of the taste cells of the galeae are responsible for sustained biting (i.e. feeding). This finding has the potential to explain host acceptance behavior by the larvae of other species of Lepidoptera and perhaps other insect orders.

Despite my enthusiasm, I have several general criticisms that I introduce here and pursue in greater detail in my specific comments. First, the interpretation of the neurophysiological mechanism of the first step of host acceptance is missing the potential role of olfaction during palpation. The design of the experiments could not determine this possibility in some cases because olfactory responses were not measured. In one other experiment in which the antennae were ablated, there is evidence that this treatment increases biting (Fig. 1), which is consistent with the hypothesis that olfactory stimuli play a role in the first step of host acceptance by deterring biting.

**Response:** Thank you very much for your kind and careful reviewing our manuscript. We appreciate the time the reviewer has spent in evaluating our work. As the reviewer pointed out, ablation of the antennae increased the number of biting during first 30 seconds in Fig. 1C but discussing a role of olfactory stimuli from this data seems very difficult. We do not agree so much with an inhibitory role of the antennae during palpation because silkworm larvae have no reasons to deter biting of mulberry leaves. Actually, as you can see in Fig. 1D, there are no differences in the timing and the number of test bites between intact and AN-ablated larvae. Therefore, we consider that the difference in the number of bites in Fig. 1C has quite a minor effect on biting behaviors and whole process of host acceptance, although unfortunately we have no idea for what increased the number of biting in AN-ablated larvae. Meanwhile, we totally agree with the reviewer’s comments that this study does not address role of olfaction in host-plant acceptance and it should be tested in detail in our future work.

**Change:** We added a description for this in lines 76-77.

Second, there are some significant gaps in scholarship, particularly from the late 1990's through the early 2000's. Many papers were published elucidating gustatory neurophysiological mechanisms of host-plant acceptance by caterpillars of several species during this time. These uncited papers are important in placing the findings of this manuscript in context. For example, Chapman 2003 (see references below) proposed a generalized gustatory neurophysiological mechanism of host-plant acceptance by phytophagous insects (especially caterpillars). The present manuscript would greatly benefit from placing its content in that context. The authors should discuss whether their findings agree with and extend the Chapman proposal in new ways or whether they argue against the Chapman proposal? I will admit that this field is not my main field, so I don't know much of the literature since the Chapman 2003 review. But I see that the review has been cited 192 times as of today, so there may be much more recent work to cite here.

**Response:** We understand the reviewer’s concern. Many papers by other workers published in last several decades have paid attention to gustatory responses of the maxillary galea as a main organ responsible for host-plant selection and overlooked the role of maxillary palp. Furthermore, most of these papers did not address contributions of these gustatory responses to feeding behavior and host-plant selection mainly because most workers did not observe the behavior itself but just recorded the outcome (e.g. feeding amounts in long-term assay). As far as we know, Chapman and Bernays were the only researchers who paid much attention to the importance of feeding behavior at the leaf surface and our findings agree with arguments in their review (Chapman and Bernays, 1989). However, even they did not notice the role of the maxillary palp. Unfortunately, we have to say that there are no conceptual advances in this field after Chapman. For that reasons, we had to cite limited papers.

The proposed model in Chapman 2003 is based on the balance of phagostimulatory and deterrent inputs sensed by the maxillary galea only. Our findings extend the model to the two-factor system by the maxillary palp and galea. Future works will address whether acceptance or rejection by sensing chemical stimuli occurs in test biting (the maxillary palp) or persistent biting (the maxillary galea) according to our model and tell true us meanings of findings by previous numerous literatures.

**Change:** We added explanation for this in the discussion section (lines 205-212).

Specific comments
Abstract, line 15. Replace "nutritional value" with "quality as food" because the food quality of a plant represents both nutritional value and effects of plant secondary metabolites. On the same line, replace "by which insects select host plants through sensing a variety of gustatory inputs" with "of host-plant selection" to avoid lengthy redundancy.

**Response & Change:** Thank you for your kind pointing it out. We modified the sentences.

Abstract, line 17. I strongly suggest replacing "authentication" with "host recognition" system. The latter is a conventional phrase that many researchers would understand. Moreover, the authors use "host recognition" elsewhere in the manuscript. There is no need to introduce a new term here. This change should be made to the title and all other parts of the manuscript.

**Response & Change:** We agree with the suggestion. Because “host recognition” indicates the first step of the two-factor system (before test biting), to include whole mechanism we found, we have replaced “authentication” with “host acceptance” along the text and in the title.

Abstract, lines 22-24. This description of the electrophysiological role of the maxillary galea is lacking much understanding about this part of the taste system, as described in various other studies and summarized in Chapman 2003. Sucrose is a critical stimulant, but secondary metabolites are key as well, with either stimulating or deterrent effects on biting and feeding.

**Response & Change:** As our main findings are a role the maxillary palp in host-plant recognition in the silkworm, we do not provide detail introduction about the electrophysiological role of the maxillary galea. We refer to it in the discussion section (lines 194-199) and we consider it enough for understanding our manuscript.

Introduction, line 30. Add "host" before "plants" for clarity, and remove "and carving their own niche" because it does not add any real information.

**Response & Change:** We modified the sentence as the reviewer pointed out.

Introduction, lines 35-37. An important reference that is missing here is a book on this topic by Chapman and de Boer from 1995 (see references).

**Response & Change:** According to the reviewer’s suggestion, we added the reference.

Introduction, lines 41 and 42. Add a hyphen, so it reads "host-plant selection." Elsewhere, this version of the phrase is used.

**Response & Change:** We added hyphens.

Introduction, lines 41-42. I would like to see a bit more of a summary of what's known and what isn't here. It's not entirely fair to say "the neuronal basis" of host-plant selection "and its roles at the leaf surface in whole host-plant selection or leaf choice are poorly understood." While there is certainly more understanding needed, this characterization of the state of knowledge is rather dismissive of what is known (some of which is described in the following paragraph).

**Response:** We are sorry for the unclear statement. In this paragraph we want to say that the leaf-surface behavior seems very important for host-plant selection but understanding of this behavior largely remains phenomenological. Many literatures using electrophysiological methods did not observe the leaf-surface behavior.

**Change:** We modified the sentences to “the neuronal basis for this behavior and to what extent the leaf-surface behavior contributes to whole host plant selection or leaf choice are poorly understood” (lines 40-42).

Introduction, line 45. Here is where the Chapman 2003 review should be cited. This paragraph should be expanded with some of the information in that paper and related ones.

**Response:** We think that extensive information about examples of chemicals that stimulate certain insects is not necessary at this stage and rather makes this introduction confusing.

**Change:** We added the citation. We modified the sentence in line 46.

Introduction, line 49. There is a grammatical issue with "number of feces." I'm not sure what the authors are trying to say here, so I can't really offer a correction.

**Response:** Thank you for your pointing it out. What we were trying to say here was that chlorogenic acid was discovered as a feeding stimulant by a long-term feeding assay in which evaluates feeding amount by counting the number of feces.

**Change:** We modified the sentence in line 49.

Introduction, lines 50-51. The end of this paragraph places this study in a very narrow context, as if everyone in the field was mostly interested in understanding host-plant selection by Bombx mori. In fact, B. mori is one of several model species used in this field, and the highly relevant work on these other species can help place the B. mori work in a broader context.

**Response & Change:** Thank you for pointing out the awkwardness. To explain it in a broader context, we added explanation at lines 49-53 as follows: “Thus, feeding stimulants and deterrents in the diet choice have been evaluated by their effects on the number of bites and feeding amounts in most cases. However, these observations do not always reflect the instantaneous behavior of insects that accepts or rejects certain plants (17) and thereby careful investigations along the leaf surface behavior is required for further understanding of role of plant secondary metabolites in host-plant selection”.

Introduction, lines 57-58. Replace "authentication" with "host recognition" and replace "that underlies oligophagy" with "responsible for the initiation of feeding" for clarity.

**Response & Change:** We replaced “authentication” with “host acceptance” as we mentioned above but did not change “that underlies oligophagy” because “the initiation of feeding” indicates just a part of the two-factor host acceptance system.

Results, line 62. Replace "behavior towards" with "of" for simplicity and clarity.

**Response & Change:** We replaced the words according to the suggestion.

Results, line 78. Replace "whether" with "how" and replace "contributes" with "contribute" for grammatical improvement.

**Response & Change:** We replaced the words according to the suggestion.

Results, line 81. The introduction of Lactuca indica here is confusing because this plant is not tested in the study reported here. I wondered if this name was a mistake because it seems like the authors might be referring to Sonchus oleraceus instead. Also in line 81, replace "consumed a relatively small amount" with "consumed in relatively small amounts."

**Response:** We are sorry for bothering you. Lactuca indica is not tested in this study due to its unavailability (instead we used Sonchus oleraceus in a biting assay) but we need to introduce the plant for the database search as you mentioned in the next comment due to unavailability of Sonchus oleraceus, conversely. So, we had to use Lactica indica alternatively.

**Changes:** To avoid confusion, we modified the text (lines 85, 86, 103, and 104). We replaced the phase according the reviewer’s suggestion (line 84).

Results, lines 101-102. Again, Lactuca indica is mentioned here, but not elsewhere.

**Response:** Please see our response to the previous comment.

Results, line 111. Replace "were" with "was."

**Change:** We replaced the word.

Results, lines 118-119. Here is where I think the possible role of olfactory inputs should be mentioned.

**Change:** We add a sentence that refers to the possible role of olfaction in the discussion section (line 182).

Results, lines 123-124. Here is where I think the broader range of stimulants should be considered for taste cells of the lateral sensilla. It might be the case that LS taste cells only respond to these sugars, but that is not the case for other caterpillar species.

**Response:** Thank you for letting us know the inaccurate sentences. As the reviewer pointed out, roles of lateral sensilla and its turning of phytochemicals are different among lepidopteran species.

**Change:** To specifically refer to the silkworm, we modified the sentences in lines 122-124.

Results, line 135. The "important role of the MG in modulating the amount of food intake" is already known from work on other species. If this information were added to the Introduction section, the statement here could say that this finding confirms "the important role…" rather than suggesting that this is a new finding.

**Response & Change:** Thank you for the suggestion. To set our finding in a precise context, we modified the phrase to “the important role of persistent biting via MG…” in line 135.

Results, line 137. Replace "in consistent" with "in agreement."

**Change:** We changed the word.

Results, line 140. Remove "that" after "whether."

**Change:** We removed “that”.

Discussion, lines 150-151. I suggest changing "comprehensively upgrade" to something like "provide new."

**Change:** We changed the words.

Discussion, line 153. Here, as in the Introduction, the state of knowledge of this field is mischaracterized. Adding the new scholarship should help the authors re-characterize the previous state of knowledge, so their contribution can be more accurately described.

**Response:** Here we refer to host selection behavior at the leaf surface and we introduced what is known for that in the second paragraph of the Introduction section.

Discussion, lines 157-161. Here is another place where I think it is important to consider the possible role of olfactory inputs in modulating the biting response. Note that the agar-based diet is not likely to have the olfactory stimuli that leaves would have.

**Response:** The suggestion is interesting and we agree that role of olfaction is next our issue to be addressed. On the other hand, silkworm larvae consumed agar-based diet including six tastants as well as mulberry leaves (Fig. 4C). This indicates olfactory inputs do not have a significant role at least in 3-h feeding assay.

Discussion, lines 164-167. I don't find this argument very convincing. In natural forests, canopy branches and leaves of multiple tree species often intersect, and individual caterpillars sometimes fall or walk off their host plant. Even dietary specialist caterpillars do not necessarily stay on the same plant for their entire larval lives. Therefore, it is likely to be important for dietary specialists of trees to have the ability to recognize their specific hosts. I agree that such specific signals do not necessarily have to be single compounds that are unique to the host, even though there are cases in which this is true (Bernays et al. 2003, see references). As argued at the end of the following paragraph, unique combinations of compounds can give specific signals, as seen in pheromone-based species recognition systems of insects.

**Response & Changes:** We agree with the suggestion and deleted this argument.

Discussion, lines 169-178. This paragraph is missing much recent scholarship (see references below) on caterpillar neurophysiological mechanisms of feeding.

**Response:** Thank you very much for the suggestion. In this paragraph we are discussing “requirements of combination of phytochemicals” based on feeding/oviposition assays but papers the reviewer had listed below does not seem to refer to “the combination”. In addition, these papers discuss the mechanism of feeding based on gustatory responses of maxillary galea they found, which may reflect partial aspects of host acceptance. For these reasons we consider we do not have to cite these papers here. Instead, we cited these papers in another paragraph discussing role of the maxillary galea.

**Changes:** With regard to numerous information on caterpillar neurophysiological response of the maxillary galea and its putative role, we added a sentence in lines194-196.

Discussion, lines 183-184. Replace "taste modalities such as sweet, bitter…" with "chemical tastants wuch as sugars, amino acids, secondary metabolites…" The former are subjective sensations, whereas my suggestion gives objective information.

**Change:** We modified the sentence as the reviewer’s comment.

Discussion, line 191. Replace "The MG has been believed to play a main role in…" with "The MG has been shown to play a main role in…" This would be a good place to cite Chapman 2003.

**Changes:** We replaced the phrase and cited Chapman 2003.

Discussion, line 193. Note that the MG in at least one species of caterpillar is known to respond to a stimulating secondary metabolite (pyrrolizidine alkaloids) at 10-12 M. Also replace "authentication" with "host recognition."

**Response:** Thank you for the information. We modified this paragraph to distinguish MG in the silkworm and other species because role of MG seems slightly different among species.

**Change:** We added this information in lines 196-199.

Discussion, line 198-199. It's also possible that olfaction plays a role in deterrent inputs provided by the MP, which contains gustatory and olfactory sensilla.

**Response:** We agree to that and “feeding deterrents” in the sentence already include olfactory inputs as well as gustatory inputs.

Discussion, lines 199-200. Replace "authentication" with "host recognition" and replace "negative selection" and "positive selection" with terms that are not conventionally understood to have different meanings in biology (evolutionary biology in this case). In this context, I suggest stimulating and deterrent effects on feeding.

**Changes:** We replaced “authentication” with “host acceptance” as explained above. And we replaced “positive selection” and “negative selection” with “stimulating effects” and “deterrent effects”, respectively.

Discussion, lines 202-206. This final paragraph reads as if the authors are struggling to place their work in a larger context. I think they will find it easier to do this when they re-frame the Introduction with additional scholarship (see references). The revised Introduction will help set up their study and the final points in the Discussion section can more clearly specify the authors' contribution in addition to specific gaps in the knowledge that future research can address.

**Response:** Thank you very for the suggestion. We agree that this paragraph was unrefined and we almost rewrote it as mentioned in response to the next comment.

Replace "authentication" in the first sentence. Later, the statement "oligophagy and polyphagy are likely consequences of the strict and loose restriction, respectively, of feeding initiation by the MP" confuses proximate and ultimate mechanisms in biology. It is almost certainly the case that the characteristics of the MP and their proximate effects on feeding behavior are a consequence of natural selection for narrow or broad diets in herbivorous insects. I suggest either rewriting this phrase or omitting it. Finally, the concluding line in the paragraph is not truly informative or helpful. At worst, it misleads the reader to believe that "mechanisms underpinning host-plant selection" are not really understood at all. In fact, there are entire books written about this topic (e.g., Bernays and Chapman, 1994, Host-plant selection by phytophagous insects).

**Response:** We deeply thank this comment for making us aware of our inaccurate statements.

**Change:** We rewrote this paragraph to set it in a precise context (lines 205-219).

Methods, line 215. Change to "Videos of silkworm feeding on leaves and filter paper…" The videos are great, by the way.

**Change:** We changed the phrase. We are glad you liked the videos.

Methods, line 218. Add "on" after "fed."

**Change:** We added the word.

Methods, line 219. Add "the" before "maxillary" and make palp, galea, and antenna plural in this sentence. Also, replace "was" with "were."

**Change:** We modified the sentences as the reviewer pointed out.

Methods, line 221. Replace "towards" with "of."

**Change:** We corrected the word.

Methods, line 223. Replace "in" with "at."

**Change:** We corrected the word.

Methods, line 225. Replace "wet" with "wetted."

**Change:** We corrected the word.

Methods, line 228. The phrase "all larvae started finally palpation towards all kinds of plant leaves" is not grammatically correct or clear. I don't know exactly what the authors are trying to say, so I don't have any correction to offer. On this line at the end, add "the" after "For."

**Response & Change:** We apologize our grammatical error. We modified the sentence to “all larvae showed palpation of all test plant leaves” in line 240. And we added “the” after “For” in the next sentence.

Methods, line 229. Add "of the" before "stimulant."

**Change:** We added the word.

Methods, line 234. Replace "investigated" with "calculated."

**Change:** We changed the word.

Methods, line 236. Add "an" before "agar-based."

**Change:** We added the precise article.

Methods, line 241. Add "the" before "agar-based."

**Change:** We added the precise article.

Methods, line 243. Provide the temperature at which the leaves were dried in the oven.

**Change:** We added the drying temperature (60°C).

Methods, line 245. Add "the" before "ratio" and replace "weight" with "mass" here and throughout this paragraph.

**Change:** We added and changed words as the reviewer pointed out.

Methods, line 252. Add "the" before "lateral" and change "sensillum" to "sensilla."

**Change:** We added and changed words as the reviewer pointed out.

Methods, line 256. Starving the larvae here and in the food intake assay can affect the results of these experiments. In addition to generalized effects of food deprivation, there can be specific effects on taste and feeding responses that reflect the insect's physiological state of nutrient deprivation. For example, dietary deficiencies in carbohydrates tend to increase the taste and feeding responses of caterpillars to carbohydrates (e.g., Bernays et al. 2004a in references). I doubt this issue has large effects on the results reported here, but I want the authors to be aware of this phenomenon in case they are not already.

**Response:** Thank you for letting us know the information. In the tip recording, the same levels of sensitivities to phytochemicals was confirmed between starved and fed larvae in our experiences. In the food intake assay, we dared to use starved larvae to loosen the restriction of feeding initiation and we cannot rule out that starvation affected the result to some extent.

Methods, line 257. The part of the line after "whereas" is incomplete.

**Change:** We completed the sentence.

Methods, line 261. Replace "being" with "it became."

**Change:** We changed the word.

Figure 1 legend, line 379. Change "persistent bites." to "persistent bites, respectively."

**Change:** We added ‘respectively’.

Figure 4. Replace "weight" in the y-axis labels of panels A, B, C with "mass." Same for the axis labels in Fig. S3.

**Change:** We modified the y-axis labels.

Figure 4 legend, line 409. Replace "weight" with "mass." Same for the legend of Fig. S3.

**Change:** We changed the words in legends of Fig. 4 and Fig. S3.

References
Bernays and Chapman. 1994. Host-plant selection by phytophagous insects. Chapman & Hall.

Chapman and de Boer, editors. 1995. Regulatory mechanisms in insect feeding. Chapman & Hall.

Bernays et al. 1998. Plant acids modulate chemosensory responses in Manduca sexta larvae. Physiological Entomology 23:193-201.

Bernays and Chapman. 2001. Taste cell responses in the polyphagous arctiid Grammia geneura: towards a general pattern for caterpillars. Journal of Insect Physiology 47:1029-1043.
A highly sensitive taste cell for pyrrolizidine alkaloids in the lateral galeal sensillum of a polyphagous caterpillar
Bernays et al. 2002. A highly sensitive taste cell for pyrrolizidine alkaloids in the lateral galeal sensillum of a polyphagous caterpillar, Estigmene acrea. Journal of Comparative Physiology A 188:715-723.

Bernays et al. 2002. A taste receptor neurone dedicated to the perception of pyrrolizidine alkaloids in the medial galeal sensillum of two polyphagous caterpillars. Physiological Entomology 27:312-321.

Bernays et al. 2003. Taste receptors for pyrrolizidine alkaloids in a monophagous caterpillar. Journal of Chemical Ecology 29:1709-1722.

Chapman. 2003. Contact chemoreception in feeding by phytophagous insects. Annual Review of Entomology 48:455-484.

Bernays et al. 2004a. Changes in taste receptor cell sensitivity in a polyphagous caterpillar reflect carbohydrate but not protein imbalance. Journal of Comparative Physiology A 190:39-48.

Bernays et al. 2004b. Gustatory responsiveness to pyrrolizidine alkaloids in the Senecio specialist, Tyria jacobaeae (Lepidoptera, Arctiidae). Physiological Entomology 29:67-72.

Rev. 2:
In this manuscript, the authors investigated the diet choice behavior of the silkworm, a famous specialist which feeds on mulberry and several close species, with different bioassay methods. They explained how mulberry preference of the silkworm was related to the appropriate combination of the three compounds in mulberry leaves. They further proposed that a two-factor authentication system, controlled by two peripheral gustatory organs of maxillary palp (MP) and maxillary galea (MG), and driven by six phytochemicals, that underlies oligophagy in silkworms. This work is interesting and provides valuable information in diet choice of phytophagous insect species. However, the conclusion is too rough to be convincing.

Assuming that combination of three compounds are responsible for mulberry preference, how to explain that silkworm larvae also feed on artificial diet (should be different compound combination even this diet contained mulberry leaves)? It was reported that polyphagous silkworm mutant strains feed on non-mulberry diets (Iizuka et al., 2012). A recent study also showed that a gustatory receptor gene determines the silkworm feeding preference (Zhang et al., 2019). In addition, possibly there are other phytochemicals in mulberry leave will also induce feeding behavior including palpation and biting. The reviewer strongly suggests that using a polyphagous mutant strain, such as Sawa-J, to make clear comparison.

**Response:** Thank you very much for your kind reviewing our manuscript. First, artificial diets contain the three compounds (CGA, ISQ, and βsito) derived from mulberry leaf powder. As you know, silkworm larvae do not consume artificial diets without mulberry leaf powder. Test biting requires quite a trace amount of CGA, ISQ/Q3R, and βsito as shown in Fig. 3C. Therefore, we consider that the host acceptance system we propose basically explains feeding of artificial diets. Indeed, it is true that we cannot rule out the possibility of existence of other phytochemicals that can induce test biting, but relevant studies for more than 60 years have identified the three compounds (CGA, ISQ, and βsito) as feeding stimulants by chemical fractionation of mulberry leaves and they are likely major feeding stimulants for silkworm larvae. Actually, the three compounds induced test biting with high probability (Fig. 3B), indicating that they are enough for host-plant recognition.

With regard to polyphagous silkworm strains, we are very sorry but we did not figure out what the reviewer was trying to suggest for improvement of this manuscript. We agree that polyphagous silkworm strains like Sawa-J are interesting materials for our further understanding of host-plant selection in the silkworm, however, comparisons between wildtype and polyphagous mutant larvae will not provide any novel concepts and valuable information on host-plant acceptance system we propose. Reasons are as follows:

First, Sawa-J larvae remain consuming mulberry leaves as well as wild-type larvae, clearly indicating that the host acceptance system is still working in the mutant. Second, Sawa-J larvae surely consume leaves from non-host plants such as cherry but feeding amounts of non-host plant leaves are just 30% in comparison to mulberry leaves (Yuzawa et al., 1991). Gr66-knockout larvae in Zhang et al. also showed quite low feeding amounts of non-mulberry diets. Accordingly, polyphagous strains do not lost preference for mulberry and, if anything, they manage to consume non-mulberry diets. Third, the mechanism of polyphagy in mutant strains is likely based on partial weakness or lack of ability to detect several feeding deterrents such as salicin and phloridzin (Iizuka et al., 2012; Asaoka, 2000). So, studies on polyphagous silkworm strains will address mechanisms of “rejection of non-host plant leaves” whereas the present study pursues the mechanism of “acceptance of host-plant leaves”.

We appreciate the reviewer’s suggestion so much. Although mechanisms of rejection of non-host plant leaves is a very interesting topic, we would like to include it in our next manuscript because it seems beyond the scope of the present study.

Yazawa et al. (1991). Feeding and gustatory responses in the “polyphagous strains” of the silkworm, Bombyx mori L. *The Journal of Sericultural Science of Japan*, 60, 363-371.

Asaoka, K. (2000). Deficiency of gustatory sensitivity to some deterrent compounds in" polyphagous" mutant strains of the silkworm, Bombyx mori. *Journal of Comparative Physiology A*, *186*, 1011-1018.

The tip recording results showed in Figure 3A and Figure S2B and C were very confusing. The concentration of chlorogenic acid (CGA), isoquercitrin (ISQ), quercetin-3-O-rhamnoside (Q3R), and β-sitosterol used in MP and MG testing were quite different. In addition, stimulus intensity is usually a positive function between stimulus concentration and the number of spikes elicited in a peripheral neuron. Thus, the authors should add the experiment of tip recording (both MP and MG) with gradient concentration. Otherwise, the present results could not support the conclusion that MP responded to the four compounds and MG did not respond to these compounds.

**Response:** According to the reviewer’s comment, we replaced tip recording data in Fig. 3A for MP with gradient concentration. The results indicate increases in the number of spikes with a dose-dependent manner, which strengthens our findings. In contrast, it is obvious that MG does not respond to chlorogenic acid, isoquercitrin, and β-sitosterol at low concentration like aM and fM because Fig. S2 clearly shows no response of MG to even high-concentrated stimulants.

**Change:** We conducted additional experiments and replaced tip recording data in Fig. 3A.

Rev. 3: Heiko Vogel - note that this reviewer has waived anonymity
This is a very nice manuscript by Tsuneto and colleagues, who have studied the perception system by which silkworm larvae decide to continuously feed (or reject) specific host plant species/plant diets.

The focus of the manuscript is on a proposed "two-factor" authentication system which larvae utilize to first perform leaf palpation, using the maxillary palp, and detecting low levels of leaf surface compounds. This first step is followed by test bites of the larvae, and includes subsequent detection of leaf sap compounds by chemosensory neurons localized on the maxillary galea and finally the induction of persistent feeding - in case the larvae encounter the correct host plant.

The authors start by illustrating what is known from the literature on decision making related to herbivorous insect feeding. Prior work from the 1960s and 1970s and more recent papers from several authors have indicated leaf surface compounds as important cues for herbivorous insects to accept host plants. In addition, numerous compounds (also mostly present on leaf surfaces) have been identified which induce oviposition of female insects. However, the major factors, insect physiology and molecular players in host plant acceptance and more generally the "turning points" in decision making of herbivorous larvae when feeding on plants were not well understood.
I found it especially interesting to see that the larval "test biting" clearly shows a frequency gradient on leaves of different host plants. In contrast to this, the persistent biting falls into two clearly separated groups: one group with the acceptable host plants show a gradient of larvae performing persistent biting and the other group (inedible leaves) display essentially zero persistent biting of larvae.

To summarize, this work does a nice job of combining multiple types of experimental approaches/assays. It is a nice finding that shows how a specialized (oligophagous) herbivorous insect can - by going through a series of "decision points" - ultimately discriminate host and non-host plants and thus accept or reject a specific plant diet. The study is well conceived and connects well with previous results, and the methodology employed in the different aspects is state of the art. The results are properly documented, and their interpretation in the discussion is quite reasonable.
I have just minor suggestions for improvement, but see no major flaws that can't be easily addressed with some additions/modifications.

Minor comments:
In all of the figures it would be helpful to provide explanations for all of the abbreviations used. Otherwise the interpretation of at least parts of the figures is severely hampered.

**Response & Change:** Thank you very much for your kind reviewing our manuscript. We added explanations for abbreviations in all figure legends.

In the discussion the authors compare their findings on compound perception, neuronal perception and decision making in larvae to adult oviposition on plant leaves. Although superficially touched upon, here or in other parts of the discussion the authors could add a few sentences on what their findings might mean in general for monophagous/oligophagous herbivores (i.e. specialist) versus highly polyphagous (i.e. generalist) ones. Do polyphagous species also require a "two-factor authentication system" - and if so what would be the main discriminatory chemical compounds. Or are polyphagous species instead rather relying on deterrents for decision making and host plant acceptance. Even though this would be rather speculative, it would provide a better approach at the more general question of what defines a oligophagous versus a highly polyphagous herbivore.

**Response:** Thank you for your suggestion. We believe that the two-factor authentication system is conserved among specialists and generalists, even beyond Lepidoptera, because this system should be based on common structures and development of gustatory organs and neuronal circuits from gustatory organs to muscles of mandibles via the suboesophageal ganglion. Prediction of discriminatory chemical compounds is difficult, but it is plausible that polyphagous insects require just single phytochemical or easy combinations for feeding initiation; instead, inhibitory effects by feeding deterrents are more important for their diet choice, as the reviewer mentioned.

**Change:** We added discussion for that in the last paragraph (lines 205-219).
