# Supplementary material for: Diet choice: The two-factor host acceptance system of silkworm larvae
Source: PLoS Biol. 2020 Sep 16;18(9):e3000828. doi: 10.1371/journal.pbio.3000828 (PMC7494105; doi:10.1371/journal.pbio.3000828)
Supplement: Attachment — Submitted filename: 200629 response to reviewers comment.docx [file pbio.3000828.s013.docx]

Response to the reviewer’s comments

Rev. 1:
This manuscript is much improved, thanks to the careful attention of the authors to the comments by the reviewers. My minor comments were addressed to my satisfaction in this revision. My major comments were mostly addressed to my satisfaction, even if I disagree with one or two responses from the authors.

For example, I am not convinced by the argument that "silkworm larvae have no reasons to deter biting of mulberry leaves" as rationale for discounting the possibility that olfactory inputs might deter biting. From an evolutionary ecology perspective, I can think of at least one reason that a caterpillar might use olfactory cues from its host plant to decide whether to feed or move elsewhere to feed. I preface my speculation with the important point that the decision to feed by a dietary specialist herbivore is not merely an automatic response to the correct host plant species (as the authors suggest in this manuscript). Insect herbivores use chemical cues to perceive phenotypic variation among individual host plants and parts of host plants, and they can respond adaptively to such variation (e.g., Knolhoff and Heckel 2014, Annual Review of Entomology 59:263). Even dietary specialist herbivores do not respond to every plant of the correct host species in the same way. For example, they might discriminate among plants with high or low resistance traits, some of which are inducible. Regarding olfactory cues, plant volatiles induced by herbivores contain information that predators and parasitoids use to locate their herbivorous prey and hosts (e.g., Heil 2014, New Phytologist 204:297), and herbivores can also use these volatile compounds emitted by plants to accept or reject host plants (e.g., Kessler and Baldwin 2001, Science 291:2141; De Moraes et al. 2001, Nature 410:577). Granted, demonstrations of this phenomenon are limited to adult moths and oviposition behavior. However, caterpillars have been shown to respond behaviorally to the induced chemistry of their host plants over very short time and spatial scales (e.g., Perkins et al. 2013, Proceedings of the Royal Society of London B 280:20122646). Even though Bombyx mori is a domesticated species, it might retain some ability to use olfactory cues from its host plant in ways that would have been adaptive for its wild ancestors.

I understand that my hypothesis for this unexplained detail in Fig. 1C is highly speculative, thus I am not asking for the authors discuss it. However, I want them to consider it for future work on the functional role of olfaction in caterpillar feeding behavior.

**Response:** We learned a lot from the comments from Reviewer 1 and deeply appreciate your kind reviewing. The comment from an evolutionary ecology perspective drops hints for further understanding of what is really happening in natural forests. We realize that “an automatic response” to phytochemicals in insect herbivores is modified by various physiological context including memory and learning. Likewise, chemistry in plants (metabolism) is also modulated by insect herbivory and other environmental stress. Needless to say, host-plant selection in natural forests is more and more complicated than we showed in this paper. However, even so, we believe that the two-factor host acceptance system is a fundamental and common mechanism to explain feeding behavior of insect herbivores.

Role of olfaction in host-plant selection is still obscure. Attraction by plant volatiles is not specific to host plants and, for example, some plant leaves attract silkworm larvae better than mulberry leaves. Meanwhile, the maxillary palp has olfactory sensilla and olfaction should be involved in leaf-surface sensing (in this case, olfaction is almost “contact chemosensation” and indistinguishable from gustation). Indeed, the olfactory role of the maxillary palp (and the antenna) is a next issue to be addressed. We speculate that larval feeding and oviposition by adult moth use common system. Thank you very much for your suggestion about future perspective.

The only revisions I would insist on at this point are some minor things to correct grammatical mistakes in sections that were re-written or added. My corrections and edits for these points are given as sticky notes on the pdf version of the manuscript attached with my review.

**Response & Changes:** According to the reviewer’s suggestion, we revised the text.
